# Bone marrow adipocytes: key players in vascular niches, aging, and disease

**DOI:** 10.3389/fcell.2025.1633801

**Published:** 2025-08-07

**Authors:** Yonggang Fan, Mai Elkhalek, Yuheng Zhang, Lu Liu, Qi Tian, Nareekarn Chueakula, Saravana K. Ramasamy, Rinkoo Dalan, Shukry J. Habib, Anjali P. Kusumbe

**Affiliations:** ^1^Tissue and Tumor Microenvironments Lab, Lee Kong Chian School of Medicine, Nanyang Technological University, Singapore, Singapore; ^2^Multidisciplinary Institute of Ageing (MIA-Portugal), University of Coimbra, Coimbra, Portugal; ^3^Lee Kong Chian School of Medicine, Nanyang Technological University, Singapore, Singapore; ^4^Department of Endocrinology, Tan Tock Seng Hospital, National Healthcare Group, Singapore, Singapore; ^5^Department of Biomedical Sciences, University of Lausanne, Lausanne, Switzerland

**Keywords:** bone marrow adipocytes, vascular niches, endothelial cell, aging, bone

## Abstract

Bone marrow adipocytes (BMAs) are emerging as metabolically active endocrine organs within the bone marrow microenvironment, engaging in extensive crosstalk with vascular niches, osteogenic cells, and hematopoietic compartments. In aging and metabolic disorders, mesenchymal and adipocyte progenitors undergo significant quantitative and qualitative transformations that shift from osteogenesis toward adipogenesis. This enhanced adipogenic profile alters the secretion of key adipokines and cytokines, thereby impairing endothelial function, destabilizing the vascular niche, and reducing hematopoietic stem cell support—culminating in bone fragility and disrupted blood cell production. Central to these alterations are pivotal signaling pathways, which orchestrate the interplay between BMAs and their surrounding cells. Furthermore, factors like oxidative stress, chronic inflammation, and endocrine dysregulation modulate BMA behavior and exacerbate their impact on marrow homeostasis. In this comprehensive review, we integrate recent advances that elucidate the molecular and cellular mechanisms underlying BMA function and their complex interactions with vascular niches. We also discuss therapeutic strategies designed to modulate BMA-mediated pathways and their downstream effects on aging and a range of diseases.

## Introduction

The bone marrow niche provides a specialized microenvironment that regulates hematopoiesis, osteogenesis, and tissue homeostasis. Within the bone marrow (BM) niche, hematopoietic stem cells (HSCs) and mesenchymal stem cells (MSCs) engage in intricate signaling crosstalk mediated by cytokines, growth factors, and extracellular matrix components. HSCs maintain hematopoietic homeostasis through tightly regulated signaling pathways such as Notch, Wnt, and CXCL12/CXCR4, which govern self-renewal, differentiation, and migration. MSCs contribute to skeletal and vascular homeostasis by differentiating into osteoblasts, chondrocytes, and adipocytes under the influence of transcription factors such as Runx2, Sox9, and peroxisome proliferator-activated receptor gamma (PPARγ) (Zhang Q. et al., 2021). Together, these cells support hematopoiesis, bone remodeling, and tissue homeostasis (Li L. et al., 2020; Aar et al., 2022). Homeostasis, differentiation, survival, quiescence, and mobilization of stem and progenitor cells are tightly regulated by the BM microenvironment within distinct vascular niches. These niches orchestrate intercellular communication via endothelial cells (ECs)-derived signaling molecules, including angiocrine factors, chemokines, and cytokines, which modulate their fate decisions. These vascular niches facilitate dynamic interactions and signaling between ECs and hematopoietic stem and progenitor cells, thereby influencing their fate. The BM vascular niche comprises three distinct ECs subpopulations: type-L, type-H, and arterial ECs (Kusumbe et al., 2014; Owen-Woods and Kusumbe, 2022; Stucker et al., 2020). The functional differences among these ECs subtypes, along with the secretion of angiogenic factors, coordinate the proliferation, differentiation, and maintenance of HSPCs, ensuring hematopoietic homeostasis. Aging-induced dysregulation of the BM vascular niche is associated with endothelial dysfunction, reduced angiogenic signaling, and increased vascular permeability, contributing to a pro-inflammatory and hypoxic microenvironment. Concurrently, aging skews MSC fate toward adipogenesis over osteogenesis via altered Wnt/β-catenin, Notch, and PPARγ signaling, exacerbated by oxidative stress and senescence-associated secretory phenotype (SASP) factors​. The progressive accumulation of adipocytes in the aging BM niche disrupts homeostasis, impairing HSC function and increasing susceptibility to hematologic malignancies. These findings suggest that both the vascular niche and the adipocytic lineage in marrow are crucial for maintaining tissue equilibrium and hematopoiesis.

Bone marrow adipocytes (BMAs) are among the most abundant mesenchymal cells in the BM, occupying up to 70% of its volume in adults (Baldelli et al., 2024). BMAs reside in the medullary cavity of long bones (e.g., tibia, femur, humerus) and vertebrae, where they contribute to regulation of bone metabolism and hematopoiesis (Piotrowska and Tarnowski, 2021). Bone marrow adipose tissue (BMAT) was first described over a century ago. Initially, BMAs were thought to be inert “space fillers” lacking significant function (Matsushita et al., 2022a; Liu et al., 2025a). Currently, BMAT plays a crucial role in various cellular and molecular mechanisms, though its precise function remains controversial. BMAT is involved in the regulation of hematopoiesis, secretion of adipokines, bone remodeling, and metabolic processes, including glucose and lipid homeostasis. Additionally, it influences systemic energy balance through factors such as leptin and angiotensin. Interestingly, although BMAT constitutes only about 10% of total adipose tissue—which includes beige (BeAT), brown (BAT), and white adipose tissue (WAT)—recent studies suggest that BMAT possesses distinct structural and functional characteristics compared to BAT and WAT (Suchacki et al., 2020). Further highlighting BMAT’s uniqueness, extensive literature characterizes BMAT as a dynamic endocrine organ distinct from other adipose depots in terms of its developmental origins, molecular markers, and physiological roles (Supplementary Text, Supplementary Table S1). BMAT undergoes lipolysis in response to metabolic stimuli, releasing free fatty acids (FFAs) through hormone-sensitive lipase and adipose triglyceride lipase activation. Additionally, BMAT exhibits reduced insulin-stimulated glucose uptake due to lower expression of glucose transporter 4 and demonstrates resistance to cold-induced glucose uptake (She et al., 2020). Under conditions such as high-fat diet (HFD) consumption, osteoporosis, anorexia nervosa, caloric restriction, altered leptin levels, and dysfunctional hematopoiesis, BMAT undergoes metabolic and molecular adaptations that alter FFAs release and affect insulin and glucose sensitivity (Piotrowska and Tarnowski, 2021; Scheller et al., 2019; Zhang et al., 2024). Alterations in BMAT function may disrupt key cellular pathways and thereby contribute to aging-related bone loss, diabetes, growth hormone deficiency, hematopoietic dysfunction, and anorexia (Wang et al., 2018; A et al., 2013). Given its unique metabolic and endocrine properties, as well as its role in supporting hematopoiesis and bone remodeling, BMAT has emerged as a potential biomarker of BM pathology. Elucidating the role of BMAT in health and disease may reveal novel therapeutic targets for BM-related metabolic and hematopoietic disorders.

Previous review literature primarily focuses on the correlation between BMAT, metabolism, and hematopoiesis (Cawthorn and Scheller, 2017; Cornish et al., 2018). This review provides a comprehensive analysis of recent findings on BMAs within the BM microenvironment and its vascular niches. We integrate the latest studies on the fundamental characteristics of BMAT and explore the regulatory factors influencing BMAT homeostasis and dysfunction. In addition, we discuss the impact of BMAT in aging, bone malignancies, and endocrine, metabolic, and immunological disorders.

## Role of BMAs during homeostasis

**FIGURE 1:**
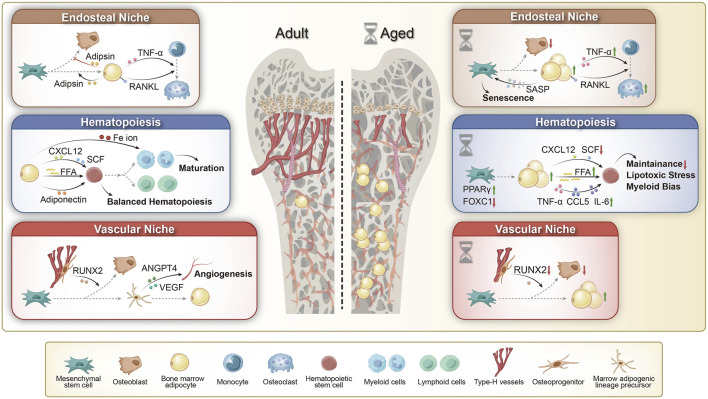
The role of BMAs in adult and aging bone marrow microenvironments. (1) Endosteal Niche: Adult: BMAs contribute to bone remodeling by secreting adipokines and enhancing RANKL and TNF-α expression, thereby promoting osteoclast differentiation and bone resorption. Aged: Aging-associated BMAs secrete senescence-associated secretory phenotype (SASP) factors, accelerating bone marrow mesenchymal stem cell (BMSC) aging. Additionally, upregulated RANKL and TNF-α signaling drive osteoclast activation and bone loss, leading to impaired bone remodeling. (2) Hematopoiesis: Adult: BMAs support hematopoiesis by secreting free fatty acids (FFAs) and adiponectin, modulating CXCL12 and SCF signaling, and regulating Fe ion availability, thereby maintaining hematopoietic stem cell (HSC) homeostasis and balanced lineage differentiation. Aged: In aging bone marrow, BMAs contribute to hematopoietic dysregulation by increasing FFA release and pro-inflammatory cytokine secretion (TNF-α, CCL5, IL-6), while downregulating CXCL12 and SCF signaling. This results in impaired HSC maintenance, increased lipotoxic stress, and a shift toward myeloid bias. (3) Vascular Niche: Adult: Type H vessels regulate bone homeostasis by supporting RUNX2-driven osteoblast activity, while bone marrow adipogenic lineage precursors (MALPs) promote angiogenesis through ANGPT4 and VEGF signaling, ensuring vascular integrity in the bone marrow microenvironment. Aged: Aging leads to BMA expansion and a decline in type H vessels, resulting in decreased RUNX2 expression and reduced osteoblast activity, thereby impairing bone homeostasis. RANKL: Receptor Activator of Nuclear Factor-kappa B Ligand; CXCL12: C-X-C Motif Chemokine Ligand 12; SCF: Stem Cell Factor; VEGF: Vascular Endothelial Growth Factor; ANGPT4: Angiopoietin-like 4; RUNX2: Runt-related Transcription Factor 2; PPAR: Peroxisome Proliferator-Activated Receptor; FOXC1: Forkhead Box C1.

BM constitutes a dynamic microenvironmental niche that plays a crucial role in regulating physiological processes throughout life. It comprises two primary niches: the endosteal and vascular niches, both of which regulate HSCs and MSCs through various signaling factors that maintain their homeostasis. However, the cellular composition of these niches may change due to aging, sex differences, and metabolic status (Zoulakis et al., 2025). HSCs give rise to myeloid progenitors that differentiate into osteoclasts via the monocyte lineage. And MSCs differentiate into adipocytes, osteoblasts, chondrocytes, and myocytes (Zinngrebe et al., 2020). By young adulthood, roughly 70% of BM volume is occupied by adipocytes. This substantial presence of BMAT influences the hematopoietic niche and metabolic regulation in the marrow (Zinngrebe et al., 2020; Veldhuis-Vlug and Rosen, 2018). BMAs and their products are integral to the normal balance between bone formation and marrow hematopoiesis, as described below (Figure 1).

### BMAs in the endosteal niche versus vascular niche

The BM niches comprise the generally well-defined endosteal and perivascular niches. The bone-marrow endosteal niche is markedly hypoxic and enriched for HIF-1α, osteopontin and Ang-1, conditions that tether haematopoietic stem cells (HSCs) in deep quiescence and couple their maintenance to osteogenesis. In contrast, the vascular niche surrounding arterioles and sinusoids is relatively oxygen-rich; LepR^+^ perivascular stromal and endothelial cells here secrete high levels of CXCL12, SCF, nitric oxide and IL-6, thereby stimulating HSC cycling, differentiation and mobilisation (Tamma and Ribatti, 2017). Bone-marrow adipocytes (BMAs), a dynamic stromal subset distributed between these regions, interact with each niche. BMAs actively regulate the endosteal niche by modulating osteogenesis (bone formation) and bone resorption. Although bone-marrow adiposity is usually inversely associated with bone mass in adult homeostasis, a transient positive coupling can emerge during highly anabolic phases such as puberty and fracture repair. In these contexts, bone marrow adipocytes exhibit a brown adipocyte-like phenotype, characterized by the expression of brown adipocyte transcription factors such as PR domain-containing 16 (Prdm16) and Forkhead box C2, as well as marker genes including PGC1α, Dio2, β3AR, and UCP1. The expression of these factors contributes to the establishment of a microenvironment that favors osteogenesis (Muruganandan et al., 2018). BMAs also secrete hormones and adipokines (e.g., leptin and adiponectin) that positively influence osteoblast proliferation and function, promoting bone formation (Zhang et al., 2024). Additionally, BMAT may protect the skeleton by sequestering excess lipids: by uptaking FFAs, BMAs can reduce lipotoxic stress on osteoblasts and prevent lipid-induced oxidative damage and apoptosis in bone-forming cells. Such protective uptake of lipids helps maintain osteoblast viability and function (Gunaratnam et al., 2014). Conversely, BMAs can inhibit osteogenesis and stimulate bone resorption under other conditions. BMAs secrete pro-inflammatory cytokines–including IL-6, tumor necrosis factor-α (TNF-α), and receptor activator of NF-κB ligand (RANKL) – that stimulate osteoclast differentiation and activity, thereby promoting bone resorption (Harmer et al., 2018). Elevated marrow adiposity is associated with higher local RANKL levels, especially in the absence of parathyroid hormone (PTH) signaling, tipping the balance toward osteoclastogenesis and bone loss (Fan et al., 2017; Kiechl et al., 2013). BMAs further enhance osteoclast formation through adipocyte-derived chemerin and sustained PPARγ activation: chemerin binding to its receptor (CMKLR1) on osteoclast precursors promotes their maturation, and PPARγ shifts MSC lineage commitment toward adipocytes (away from osteoblasts), indirectly favoring bone resorption by reducing new bone formation (Muruganandan and Sinal, 2014). Collectively, these interactions position BMAs as key regulators of bone remodeling. Under homeostatic conditions, BMAs can support osteogenesis through anabolic signaling pathways, whereas in aging or osteoporosis their accumulation skews the niche toward adipogenesis and bone breakdown, contributing to loss of bone mass. This dualistic role highlights the complex influence of BMAs on skeletal homeostasis.

The bone marrow vascular niche–composed of a network of type-H vessels and sinusoids type-L vessels–provides crucial cues for mesenchymal lineage allocation and indirectly affects BMA development (Chen et al., 2020). Signals from vascular-associated cells can regulate BMA formation. Perivascular cells such as pericytes, vascular smooth muscle cells, and CXCL12-abundant reticular (CAR) cells have been implicated in promoting adipogenesis within the BM vascular microenvironment. The two major vessel types in BM have distinct effects on mesenchymal lineage allocation. Type-H endothelial cells mediate local vascular growth and provide niche signals that promote the expression of osteogenic transcription factors such as Osterix and Runx2 in adjacent perivascular osteoprogenitors, thereby directing nearby MSCs to become osteoblasts rather than adipocytes (Kusumbe et al., 2014). In contrast, type-L vessels lack these osteogenic signals and are enriched in CAR cells and leptin receptor-positive (LEPR^+^) stromal cells that secrete stem cell factor (SCF) and CXCL12. SCF and CXCL12 are not only vital for HSC maintenance but also create a microenvironment that can support adipocyte differentiation. CAR cells directly promote adipogenesis via PPARγ signaling (Omatsu et al., 2014); accordingly, broad ablation of CXCL12^high^ CAR cells leads to a marked reduction in marrow adipocytes and can perturb osteogenesis and HSC maintenance (Omatsu et al., 2010), whereas selective deletion of the Adipoq^+^ CAR subset instead enhances bone formation (Zou et al., 2025). Other Nestin^low^ perivascular progenitors (characterized by high PDGFR-α/β expression) similarly give rise to BMAs, underscoring that the vasculature serves as a niche for adipocyte progenitors (Jiang et al., 2021). Interestingly, lineage-tracing studies suggest that in blood vessel walls outside the marrow, only adventitial fibroblasts (in the outer vessel lining) have significant adipogenic potential, whereas ECs and mural cells do not contribute to new adipocytes (Cattaneo et al., 2020). It remains to be seen whether analogous adventitial cells in BM vessels are a source of BMAs. Overall, these findings illustrate that the vascular niche provides important developmental cues for BMAs.

BMAs in turn actively contribute to the vascular niche and local blood supply. BMAs secrete a diverse array of adipokines, cytokines, growth factors, and other mediators that can influence ECs and blood vessel function. For example, a recently identified subset of BM adipocytes with low lipid content supports angiogenesis by secreting vascular endothelial growth factor (VEGF) and angiopoietin-like 4. These factors promote blood vessel growth and stability, helping to maintain the vascular network of the marrow and its capacity to support hematopoiesis (Wang et al., 2021; Zhong et al., 2020). In general, BMAs also serve as an energy reservoir for the niche: their lipid droplets (rich in saturated, monounsaturated, and polyunsaturated fatty acids) can be mobilized to provide fatty acids as fuel for neighboring cells, including osteoblasts, osteoclasts, and HSCs. Additionally, BMAs secrete adiponectin, an anti-inflammatory adipokine that improves insulin sensitivity and lipid metabolism through AMPK and PPARα activation. Adiponectin released into the marrow vasculature may help dampen local inflammation and thereby benefit the vascular niche (Ouchi and Walsh, 2007). In sum, BMAs are now recognized as integral components of the vascular niche, helping to maintain vascular homeostasis and angiogenesis in the BM microenvironment.

### Signaling pathways regulating BMAs

The differentiation and function of BMAs are governed by a complex network of molecular signals, integrating local niche-derived factors and systemic hormonal cues (Table 1). A delicate balance exists between adipogenesis (formation of adipocytes) and osteogenesis (formation of osteoblasts) in the BM, largely regulated by opposing pathways. On one side, PPARγ is the master transcription factor driving adipocyte differentiation; on the other, Wnt/β-catenin signaling promotes osteoblast commitment and suppresses adipogenesis. The BM microenvironment leverages these pathways to modulate BMAs. For example, in the absence of strong osteogenic cues, MSCs more readily undergo adipogenesis under the influence of PPARγ together with C/EBPα and other adipogenic factors (such as fatty acid-binding protein 4 [FABP4] and perilipin-2). Several paracrine factors in the endosteal niche also influence this balance (Nuttall et al., 2014; Liu et al., 2011). Osteoclasts secrete semaphorin 4D, which binds to osteoblast precursors (through Plexin-B1 receptors) to potently inhibit osteoblast differentiation while simultaneously enhancing adipogenic differentiation of MSCs (Muruganandan and Sinal, 2014; Wan, 2013; Ishii et al., 2022). This coupling of bone resorption activity to fat formation ensures that increased osteoclast activity (as in low estrogen or inflammatory states) can lead to greater BMA accumulation. Conversely, osteoblast-lineage cells produce semaphorin 3A (Sema3A), which has the opposite effect: Sema3A signaling via neuropilin-1 promotes osteogenesis and suppresses adipocyte and osteoclast differentiation, thereby protecting bone mass​. Similarly, factors like Delta-like 1 (Dlk1) must be downregulated for adipogenesis to proceed; Dlk1 (also known as Pref-1) normally inhibits preadipocyte differentiation, so its absence greatly enhances adipocyte formation (while somewhat paradoxically also permitting increased osteoblast differentiation through upregulation of pro-osteogenic genes like Runx2) (Lin et al., 2019; Tencerova and Kassem, 2016). Inflammatory cytokines present in the BM niche can further tip the balance: for instance, Oncostatin M and IL-6 released during inflammation have been shown to stimulate osteoblast generation at the expense of adipocytes, at least in short-term settings (West et al., 2018). Through these local signaling mechanisms, the BM niche tightly coordinates the inverse relationship between bone formation and marrow adiposity.

**TABLE 1:** The role of bone marrow adipocytes.

Category	Function	Factors/Signal pathway	Mechanism	Condition	Reference
Endosteal niche (Bone remodeling)	Osteogenesis inhibition	PPARγ activation	Drive adipogenesis, Suppress osteoblast lineage	Aging, Osteoporosis, Metabolic disorders	PMID: 27012163
Endosteal niche	Osteogenesis inhibition	Wnt/β-catenin inhibition	Block osteoblast differentiation, Reduce bone formation	Bone remodeling imbalance, Osteoporosis	PMID: 29855795
Endosteal niche	Osteogenesis inhibition	Lipotoxicity (palmitate, FFAs)	Release saturated lipids, Induce osteoblast apoptosis	Obesity, Aging, High-fat diet	PMID: 24169557
Endosteal niche	Osteogenesis inhibition	Glucocorticoid-induced suppression	MSC fate shift to adipocytes, Wnt signaling downregulation	Glucocorticoid therapy, Osteoporosis	PMID: 28293453
Endosteal niche	Osteogenesis inhibition	Estrogen deficiency	Increase PPARγ, Inhibit osteoblast function	Menopause, Postmenopausal osteoporosis	PMID: 37682419
Endosteal niche	Promotes bone resorption	RANKL-NFκB pathway	Induce osteoclast formation, Enhance bone resorption	Aging, Osteoporosis	PMID: 23396210
Endosteal niche	Promotes bone resorption	Chemerin-CMKLR1 axis	Stimulate osteoclast precursors, Boost resorption	Bone loss, Adipocyte-driven inflammation	PMID: 24638917
Endosteal niche	Promotes bone resorption	Pro-inflammatory cytokines	Increase osteoclastogenesis, Exacerbate inflammation	Chronic inflammation, Metabolic disorders	PMID: 30671025
Endosteal niche	Promotes bone resorption	Glucocorticoid-enhanced osteoclastogenesis	Prolong osteoclast lifespan, Suppress osteoblasts	Steroid use, Osteoporosis	PMID: 22870429
Endosteal niche	Promotes bone resorption	Bone malignancies	Elevate IL-6/RANKL, Induce osteolytic lesions	Multiple myeloma, Leukemia, Bone metastases	PMID: 30671025
Endosteal niche	Promotes bone resorption	Hyperglycemia, oxidative stress	Upregulate NFκB, Amplify osteoclast activity	Diabetes-associated bone loss	PMID: 31551934
Endosteal niche	Potential protective effects	Lipid buffering effect	Sequester excess FFAs, Reduce lipotoxic damage	Certain metabolic states, Mild adipogenesis	PMID: 24169557
Endosteal niche	Potential protective effects	Hormones and adipokines	Enhance osteoblast proliferation, Promote bone formation	Skeleton protection	PMID: 31734905
Hematopoiesis (HSC niche)	HSC maintenance, Self-renewal	SCF/CXCL12 axis	Maintains HSC quiescence, Enhances HSC regeneration	Normal, Hematopoiesis, Stress recovery	PMID: 34912805 PMID: 24590069​
Hematopoiesis	HSC maintenance, Self-renewal	Lipid metabolism, FFA release	Store and release FFAs, Fuel HSC under stress	Starvation, Chemotherapy, Irradiation recovery	PMID: 31890207
Hematopoiesis	HSC differentiation, Lineage commitment	PPARγ-driven BMAT expansion	HSC shift to myelopoiesis, Enhanced granulocyte, Monocyte production	Aging, Inflammation, Metabolic disorders	PMID: 35360075​
Hematopoiesis	HSC differentiation, Lineage commitment	IL-6/TNF-α/CCL5-mediated inflammatory signaling	Promote myeloid bias, Reduce lymphopoiesis	Age-related immune dysfunction, Chronic inflammation	PMID: 31806690
Hematopoiesis	HSC differentiation, Lineage commitment	β2-AR activation	Promote myeloid expansion, Reduce lymphoid survival	Aging, Stress, Immunosenescence	PMID: 31303548
Hematopoiesis	Impact on HSC niche	Foxc1 suppression, Adipogenic niche replacement	Replace stroma with adipocytes, Disrupt HSC support	BMAT expansion, Loss of osteogenic niche	PMID: 24590069
Hematopoiesis	Impact on HSC niche	Cholesterol transport deficiency (ABCA1/G1 dysfunction)	Impair cholesterol efflux, Alter HSC fate	Aging, Lipid metabolism disorders	PMID: 20488992
Hematopoiesis	Hematopoietic recovery, Malignancies	BMAT-derived CXCL12, leptin	Fuel malignant cells, Enhance tumor survival	Leukemia, Multiple myeloma, Therapy resistance	PMID: 27863383
Hematopoiesis	Hematopoietic recovery, Malignancies	FABP4-mediated FFA transport in AML	Transfer FFAs to AML, Increase chemoresistance	AML, Metabolic, Adaptation of cancer cells	PMID: 28049638
Hematopoiesis	Hematopoietic recovery, Malignancies	Decreased GATA2 expression	Excessive BMAT accumulation, Impair MSC differentiation	Aplastic anemia	PMID: 24876847
Vascular niche (Angiogenesis)	Regulate vascular, Endothelium	SPTBN1-VEGF	Stimulate endothelial proliferation, Support vessel formation	Normal, Angiogenesis, Fracture repair, Wound healing	PMID: 33816505
Vascular niche	Interaction with BMAs	BMAT-derived VEGF, ANGPTL4	Promote local vessel growth, Stabilize endothelium	Bone marrow vascular niche, Steady-state hematopoiesis	PMID: 34912805
Vascular niche	Interaction with BMAs	Adiponectin (anti-inflammatory)	Improve endothelial function, Reduce vascular inflammation	Obesity, Anti-inflammatory environment	PMID: 17343838
Vascular niche	Regulate vascular aging, Dysfunction	Oxidative stress (ROS)	Endothelial dysfunction, Pro-inflammatory environment	Diabetes, Metabolic syndrome, Chronic inflammation	PMID:38727260
Metabolic regulation (Energy homeostasis)	Systemic energy balance	PPARγ activation	Drive adipocyte differentiation, Influence lipid storage	Obesity, Aging, Glucocorticoid therapy	PMID: 22863012
Metabolic regulation	Systemic energy balance	Leptin secretion	Regulate appetite, Modulate bone-fat axis	Normal nutritional status, Leptin resistance	PMID: 27053299
Metabolic regulation	Systemic energy balance	Adiponectin	Enhance insulin sensitivity, Reduce inflammation	Caloric restriction, Anti-inflammatory conditions	PMID: 14699128
Metabolic regulation	Systemic energy balance	BMAT lipolysis, FFA release	Provide metabolic substrates, Support peripheral tissues	Starvation, Intense exercise, Chemotherapy	PMID: 31890207
Metabolic regulation	Cross-talk with adipocyte types	WAT-derived adipokines	Impact glucose metabolism, Affect BMAT function	Obesity, Insulin resistance	PMID: 30038881
Metabolic regulation	Cross-talk with adipocyte types	BAT-related thermogenesis	Regulate marrow adipocyte browning, Energy dissipation	Cold exposure, Metabolic interventions	PMID: 21135534
Metabolic regulation	Cross-talk with adipocyte types	Endocrine signals	BMAT Formation, Systemic Energy Regulation	GH deficiency, Cushing’s syndrome	PMID: 19821771
Metabolic regulation	Metabolic disorders, Aging	Insulin resistance	Decreased GLUT4 expression, Impaired glucose uptake	Type 2 diabetes, Obesity	PMID: 32778749
Metabolic regulation	Metabolic disorders, Aging	Excess glucocorticoids	Facilitate BMAT accumulation, Dampen osteoblastogenesis	Cushing’s syndrome, Prolonged steroid therapy	PMID: 36564571
Metabolic regulation	Metabolic disorders, Aging	Aging-related lipotoxicity	Saturate FFA accumulation, Marrow ROS elevation	Osteoporosis, Immunosenescence	PMID: 24169557
Metabolic regulation	Potential therapeutic targets	TZDs (PPARγ agonists)	Improve insulin sensitivity, Expand BMAT	Type 2 diabetes, Metabolic syndrome	PMID: 22304921
Metabolic regulation	Potential therapeutic targets	Nicotinamide riboside, Sirt1 activators	Boost mitochondrial function, Attenuate adipogenic shift	Aging, Diabetic bone complications	PMID: 31000692
Immune modulation (Inflammatory environment)	Basal immune regulation	Adiponectin (anti-inflammatory)	Lowers NF-κB activity	Mild inflammation, Calorie restriction	PMID: 27529061
Immune modulation	Basal immune regulation	Leptin	Activates T cells	Obesity, Leptin resistance	PMID: 27863383
Immune modulation	Basal immune regulation	Adipsin	Enhances insulin secretion, Impacts complement cascade	T2DM management, Adipokine interplay	PMID: 27863383
Immune modulation	Secrete cytokines, Immune cell differentiation	CCL5 (RANTES)	Recruit monocytes, Foster myeloid cell expansion	Obesity-associated inflammation, Atherosclerosis	PMID: 22289892
Immune modulation	Secrete cytokines, Immune cell differentiation	Functional defects in BMAT-derived MSCs	Alters MSC immunomodulatory function	RA flares, Chronic joint inflammation	PMID: 35386146
Immune modulation	Secrete cytokines, Immune cell differentiation	Decreased BMAT levels	Increased IL-7 and neutrophils, Immune environment shift	SpA pathogenesis, Enthesitis	PMID: 34149700

Beyond the local microenvironment, systemic and environmental factors profoundly influence BMA formation and function. Below, we outline BMA dynamics under conditions of obesity, caloric restriction, and circadian disruption.

### BMA dynamics during obesity, hormone treatment, fasting, circadian rhythm

A HFD and obesity are associated with increased BM adiposity. Although circulating leptin levels rise in obesity, bone marrow MSCs often develop leptin resistance, leading to impaired JAK2/STAT3 signaling. This favors PPARγ-driven adipogenesis and suppresses osteoblastogenesis. Consequently, obese individuals often exhibit higher marrow fat content and reduced bone formation. Nutritional excess is, therefore, a driver of BMAT expansion, whereas calorie restriction tends to limit leptin signaling (though extreme cases like anorexia can paradoxically increase BMAT, as noted below) (Yue et al., 2016).

Glucocorticoids (stress hormones or therapeutic steroids) strongly induce marrow adipogenesis; patients on long-term glucocorticoid therapy often show elevated BMAT and concurrent bone loss. Conversely, estrogen has protective effects against BM fat accumulation–estrogen deficiency (for example, after menopause) is linked to a surge in BMAs along with osteoporosis (Li et al., 2024). Insulin-like growth factor 1 (IGF-1) and thyroid hormones are additional systemic factors that modulate BMAT homeostasis. From a therapeutic standpoint, targeting JAK2/STAT3, a pathway implicated in certain pathological BMAT expansions (e.g., leukemia-associated), has shown promise: in a chronic lymphocytic leukemia model, inhibiting JAK2/STAT3 reduced pathological marrow adipogenesis more effectively than dietary changes, highlighting a potential intervention to rebalance the niche (Severin et al., 2019).

Paradoxically, conditions of extreme energy deficit, such as prolonged fasting or anorexia nervosa, are associated with increased marrow adiposity. In these states, peripheral fat is depleted and BMAT may serve as a last reservoir of energy, or the lack of nutrients may trigger stress signals (e.g., high cortisol) that stimulate adipogenesis in the marrow. Thus, the relationship between systemic energy balance and BMAT is bidirectional and context-dependent–acute fasting reduces leptin (removing an anti-adipogenic signal) yet other factors can drive adipocyte accumulation as a survival mechanism (Piotrowska and Tarnowski, 2021).

The BM niche is under circadian control, and disrupting normal day-night cycles can alter adipocyte formation. Core clock genes such as CLOCK, BMAL1, and PER2 regulate adipogenic signaling pathways (Hafidi et al., 2019). Notably, BMAL1 (a key circadian regulator) promotes adipocyte differentiation, and cross-talk with PPARα/γ has been observed in regulating lipid metabolism and circadian rhythms (Aggarwal et al., 2017). Therefore, disturbances in circadian rhythm (due to shift work, jet lag, or sleep disorders) can lead to abnormal BMAT expansion. Studies have shown that knocking out BMAL1 reduces marrow adipogenesis, while circadian disruption increases it (Kawai et al., 2010a; Kawai et al., 2010b; Oishi et al., 2008; Fairfield et al., 2021). In line with this, experimental models demonstrate that alteration of clock genes or sleep patterns results in imbalanced adipogenic signals in the BM microenvironment.

In homeostasis, these regulatory mechanisms ensure a balanced interplay between bone, fat, and hematopoiesis in the marrow. With aging or metabolic disease, shifts in signaling (e.g., heightened PPARγ activity, chronic inflammation, or endocrine changes) lead to disproportionate BMAT expansion and associated dysfunctions in bone and blood cell production. Conversely, in pathological conditions such as leukemia, malignant cells can exploit normal signaling pathways (like leptin-JAK/STAT or CXCL12-CXCR4) to favor an adipocyte-rich, tumor-supportive niche. By unraveling the molecular regulation of BMAs, we can better understand their versatile roles in steady-state physiology, ultimately guiding interventions for bone marrow disorders.

## Role of BMAs during hematopoiesis

BMAs profoundly influence hematopoiesis (blood cell formation) through both direct and indirect mechanisms. Under steady-state conditions, BM adipocyte-lineage cells, particularly adipocytic progenitors, contribute to the hematopoietic niche by creating a supportive environment for HSCs and their progeny. One key role of BM adipocytes is in regulating nutrient and growth factor availability for blood-forming cells. Adipocyte-lineage stromal cells are a source of SCF and CXCL12, two crucial niche factors that promote HSC maintenance, quiescence, and regeneration (Wang et al., 2021; Zhong et al., 2020; Shafat et al., 2017; Mattiucci et al., 2018). The release of FFAs from BMAs can provide metabolic fuel to highly proliferative hematopoietic cells; in fact, adipocyte-derived FFAs have been shown to support HSC recovery following stress and to enhance HSC progenitor function (Pernes et al., 2019). Through these secreted factors and nutrients, BMAs help sustain hematopoietic stem and progenitor cells, contributing to normal blood cell homeostasis. Consistently, moderate BMAT is a feature of healthy marrow, and its adipokines (like adiponectin) may help maintain an anti-inflammatory milieu that is conducive to balanced hematopoiesis (Baldelli et al., 2024; Scherer, 2014). Overall, recognizing the dual nature of BMAs in hematopoietic regulation is crucial for developing therapies for bone marrow failure or malignancies that consider the adipocytic component of the niche.

While a direct causal link between increased BM fat and HSC myeloid bias is still under investigation, there is evidence suggesting that excessive BMAT can contribute to skewed hematopoiesis. High levels of BM adiposity have been correlated with a tendency toward myeloid-biased HSC differentiation (Baldelli et al., 2024; Aguilar-Navarro et al., 2020). Furthermore, disruption of critical niche factors can mimic aging effects: for example, deleting the transcription factor Foxc1 (which, as noted, normally suppresses adipogenesis and supports HSC niches) leads to replacement of hematopoietic cells with adipocytes and a significant loss of HSCs (Omatsu et al., 2014; Nakamura-Ishizu and Suda, 2014). This Foxc1 deletion model illustrates how increased marrow fat can directly displace hematopoiesis. Metabolic changes in the marrow may also play a role—impaired lipid handling in the HSC niche (e.g., deficient cholesterol efflux) has a negative impact on HSC function. Notably, efficient cholesterol efflux via transporters ABCA1/G1 and high-density lipoprotein is necessary to restrain HSC proliferation; disruptions in these pathways can lead to aberrant HSC expansion or exhaustion (Aar et al., 2022; Yvan-Charvet et al., 2010). It is not yet clear if the presence of excess BM adipocytes directly drives such metabolic perturbations or if they arise from parallel age-related changes in the niche. Another BMA-related alteration in HSC biology involves the plasticity of marrow adipocytes themselves. Recent studies show that, following irradiation or chemotherapy, BMAs emerges and upregulates SCF, thereby accelerating endothelial repair and facilitating HSC re-engraftment (Hirakawa et al., 2023; Zhou et al., 2017). Lineage-tracing experiments further demonstrate that mature BMAs can dedifferentiate into LepR^+^ cells, revealing a BMA-to-LepR^+^ cells plasticity that is indispensable for haematopoietic recovery; conversely, genetic blockade of adipocyte lipolysis (Atgl deletion) abolishes this dedifferentiation and dramatically impairs haematopoietic regeneration (Hirakawa et al., 2023). Collectively, these findings expand the functional repertoire of BMAs—from passive energy reservoirs to reversible niche cells that actively coordinate vascular and haematopoietic repair under stress.

### BMAs in aging

Aging is associated with increased risk of many chronic diseases (e.g., cardiovascular disease, diabetes, osteoporosis) due to cellular senescence, mitochondrial dysfunction, oxidative stress, and chronic inflammation (Blume and Curtis, 2011; Chen et al., 2021). One hallmark of aging is reduced regenerative capacity of tissues caused by a decline in stem cell function. In bone marrow, aging leads to a progressive accumulation of BMAs at the expense of osteogenesis, as MSCs increasingly differentiate into adipocytes rather than osteoblasts (Liu ZZ. et al., 2021). This expansion of BM fat is associated with disruption of the bone marrow microenvironment, impairing both MSCs and HSCs function and disturbing the delicate balance between adipogenesis and osteogenesis (Alt et al., 2012) (Figure 1). Age-related deterioration of the marrow niche also involves vascular changes, including a decline in type-H endosteal blood vessels that normally support osteogenesis and hematopoiesis​. Loss of these vessels exacerbates local hypoxia and oxidative stress, further damaging ECs and osteoprogenitors (Kusumbe et al., 2014; Wang et al., 2017a). Consequently, aged bone marrow ECs produce lower levels of HSC-supportive factors (e.g., CXCL12, SCF, and Notch ligands). These changes contribute to hematopoietic imbalance, immune dysregulation, and bone loss with age (Poulos et al., 2017; Kusumbe et al., 2016).

MSCs in aged bone marrow undergo a functional decline characterized by reduced proliferation and a diminished capacity to form bone, coupled with an enhanced propensity to form fat. This age-induced shift in lineage commitment—often termed the adipogenic switch—results in a gradual “yellowing” of the marrow as adipocytes accumulate at the expense of osteoblasts. Aged MSCs show molecular signs of senescence, including increased ROS and upregulation of cell-cycle inhibitors (p53, p21, p16^INK4a^), leading to proliferation arrest. Accumulation of advanced glycation end-products in the bone matrix further drives this process by inducing oxidative stress and apoptosis in MSCs (Wan et al., 2023). In addition, aged MSCs accrue DNA damage (e.g., telomere shortening and epigenetic alterations), which undermines their regenerative potential (Zheng et al., 2013; Zhang et al., 2011; Kornicka et al., 2015; Ju et al., 2007). These intrinsic changes bias MSC differentiation toward adipogenesis over osteogenesis, thereby promoting BMA expansion in the aging marrow.

In addition to intrinsic changes in aged MSCs, extrinsic factors in the aging niche also enforce the adipogenic switch. For example, age-related increases in Wnt pathway inhibitors like Dickkopf-1 (DKK1) and sclerostin lead to reduced Wnt signaling, which impairs osteoblast differentiation and favors adipocyte formation (Colditz et al., 2020; Fairfield et al., 2018; Himburg et al., 2017). Chronic inflammation in aging bone marrow elevates cytokines that suppress osteopontin (OPN) expression in MSCs. Since OPN normally inhibits adipogenesis (Scutera et al., 2018), its reduction removes a brake on fat formation, further skewing MSCs toward the adipocyte lineage (Chen et al., 2014). In peripheral adipose tissue, OPN deficiency is associated with reduced adipose tissue macrophages (ATMs), which regulate norepinephrine-driven lipolysis; whether a similar OPN–ATM–lipolysis axis exists in bone-marrow adipose tissue remains to be established (Camell et al., 2017; Guidi et al., 2017).

In addition, several key molecular regulators become dysregulated in aged MSCs, tipping the balance toward adipogenesis. The transcription factor FOXP1, which normally interacts with C/EBPβ/δ and Notch signaling to promote osteogenesis and restrain adipogenesis, is reduced with aging (Li et al., 2017). In mice, loss of FOXP1 causes an osteoporosis-like phenotype with increased BMAT, lower bone mass, and impaired MSC self-renewal (Li et al., 2017). MicroRNAs are another regulatory layer: miR-188, for instance, is upregulated in aged MSCs and drives adipocyte differentiation while suppressing osteoblast formation, contributing to bone loss (Li et al., 2015). Conversely, miR-130a levels decline with age, leading to elevated PPARγ (a master adipogenic factor) activity and further promotion of adipogenesis at the expense of osteogenesis (Lin et al., 2019). Aging is also accompanied by the decline of supportive niche cells and signals that normally help maintain the osteogenic capacity of the bone marrow (Omatsu et al., 2014; Galan-Diez and Kousteni, 2018). Perivascular MSC populations (such as those lining blood vessel walls) and CAR cells decrease in number with age. These stromal cells produce factors like CXCL12, SCF, and Foxc1 that support HSC maintenance while simultaneously suppressing adipocyte differentiation. With advanced age, the loss of bone marrow pericytes (particularly those associated with type-H vessels) leads to reduced levels of quiescence-promoting signals (CXCL12, PDGF-β, SCF), undermining the HSC niche and allowing more adipogenic differentiation of MSCs (Singh et al., 2019; Supakul et al., 2019). The loss of perivascular PDGF-β signaling disrupts the bone marrow niche, facilitating the proliferation of disseminated tumor cells while promoting adipogenic differentiation at the expense of osteogenesis (Glatt et al., 2007; Justesen et al., 2004). Similarly, aging marrow exhibits diminished Notch signaling; normally, marrow ECs present high levels of Notch ligand 2 to nearby stromal cells to promote osteogenesis, but this signal wanes with age, tilting MSC fate toward adipogenesis (Guo et al., 2017; Sacma et al., 2019; Bi et al., 2016). Together, these intrinsic and extrinsic age-related changes orchestrate a shift toward greater marrow fat deposition and fewer bone-forming cells.

### Hematopoiesis changes

BMAs play a context-dependent role in hematopoiesis: they are important supportive cells in normal BM physiology, but in aging, their proliferation and secretory profile shift in ways that are associated with impaired hematopoiesis and may contribute to hematopoietic disorders. The expansion of BM adipocytes with age has profound effects on hematopoiesis. Elderly individuals commonly experience hematopoietic dysfunction, such as anemia and weaker immune responses. Studies show that increased BMAT is associated with a decline in both the number and functional capacity of HSCs, contributing to impaired blood cell production in aging marrow (Veldhuis-Vlug and Rosen, 2018; Mendelson and Frenette, 2014; Ho and Mendez-Ferrer, 2020). In normal (young) bone marrow, a baseline level of BM adipocytes supports hematopoiesis by secreting key factors. One crucial adipocyte-derived factor is SCF: BMAs secrete SCF, which binds to c-Kit on HSCs to promote HSC survival and proliferation (Zhou et al., 2017). Consistently, Adipoq-Cre–mediated deletion of Scf—which removes SCF from both BMAs and other Adipoq^+^ stromal populations such as LepR^+^/CXCL12^high^ CAR cells—results in a marked reduction in HSC numbers and myeloid progenitors in middle-aged mice (Zhang et al., 2019; Jeffery et al., 2022). These findings confirm that BMAT-derived SCF is essential for maintaining steady-state hematopoiesis and is crucial for supporting HSC function under metabolic stress during aging.

A hallmark of aged HSCs is a bias toward myeloid lineage output at the expense of lymphoid lineages (Geiger et al., 2013). In other words, aging skews blood production toward neutrophils and monocytes rather than B and T lymphocytes. Chronic low-grade inflammation in the aged marrow niche appears to drive this myeloid skewing; for instance, the pro-inflammatory chemokine CCL5 is elevated in aging and pushes HSC differentiation toward myeloid cells. CCL5 also affects the bone marrow microenvironment by influencing bone remodeling and HSC niche signaling, linking inflammatory changes to HSC fate decisions (Aar et al., 2022; Ho and Mendez-Ferrer, 2020; Budamagunta et al., 2021). Vascular ageing and reduced endothelial nitric oxide synthase (eNOS/NOS3) activity remodel the BM vasculature and are accompanied by myeloid-skewed hematopoiesis, recapitulating an ageing-like niche (Ho et al., 2019). Aging is associated with alterations in adrenergic receptor (AR) signaling, where β2-AR activity, unlike β3-AR, plays a role in promoting myeloid skewing. The loss of β3-AR activity disrupts endosteal niches, leading to a reduction in osteoblast-supportive regions, which puts HSCs in further proximity from the endosteum and induces myeloid bias over lymphopoiesis (Ho et al., 2019). The accumulation of aged HSCs and increased β2-AR activity contribute to the expansion of central bone marrow capillaries, myeloid cells, and megakaryocytes (Ho and Mendez-Ferrer, 2020). Consistent with these changes, aged bone marrow shows an expansion of central marrow blood vessels, myeloid cells, and megakaryocytes.

### BMAs phenotypic changes

While some studies have focused on endosteal and vascular niche factors, others have investigated the molecular mechanisms underlying BMAT regulation during aging. Compared with peripheral white fat, BMAT expresses lower levels of mature adipocyte markers (PPARγ, FABP4, PLIN1, adipsin) yet retains relatively high transcripts of early adipogenic regulators, including C/EBPβ and RGS2. This pattern reflects an immature, stress-adapted phenotype. BMAT expresses IL-6 and GPR109A more strongly than WAT at baseline, yet both transcripts decline as animals age (Liu et al., 2011; Liu et al., 2013). Analysis of bone and adipose tissues in lipodystrophic “fat-free” mice during aging has revealed a novel secondary adipogenesis pathway that is activated under metabolic stress and aging conditions. This process involves the recruitment of adiponectin-negative stromal progenitors, which differentiate into BMAs independent of traditional PPARγ-driven pathways. This BMAT exhibits impaired lipid mobilization and altered cytokine expression within hematopoietic regions. Notably, it is characterized by low levels of adiponectin and CXCL12 and demonstrates resistance to lipolytic stimuli. These findings provide new insights into the mechanisms underlying BMAT adaptation and its potential impact on the bone marrow microenvironment (Matsushita et al., 2022b).

Notably, some interventions can counteract the adipogenic shift in aged marrow. Supplementation with nicotinamide mononucleotide activates SIRT1-dependent pathways in MSCs, which promotes osteogenic differentiation while suppressing adipocyte formation (Song et al., 2019). Enhancing autophagy in MSCs via the receptor optineurin has also been shown to reduce adipogenesis by clearing pro-adipogenic factors like FABP3 (Liu ZZ. et al., 2021). Even the pharmacological reduction of BMAT is possible: for example, dipeptidyl peptidase-4 (DPP4) inhibitors were found to reverse BMAT-induced suppression of bone healing (Ambrosi et al., 2017). These findings underscore that age-related marrow fat accumulation is not irrevocable and can be therapeutically targeted to improve bone formation in the elderly.

### Impact of BMAs on osteogenesis and bone health

Age-related BMA accumulation not only impairs blood formation but also undermines bone formation. The presence of excessive adipocytes in marrow alters the niche in ways that inhibit osteoblast function and bone regeneration (Ambrosi et al., 2017). One contributing factor is the dysregulation of Wnt signaling: as mentioned above, aged marrow fat cells produce factors that antagonize Wnt, a pathway crucial for osteoblast maturation. Elevated levels of pro-inflammatory cytokines from BMAs can also suppress osteoblast activity, leading to reduced bone formation and accelerated bone loss (Clabaut et al., 2021). Studies have shown that adipocyte-secreted factors directly downregulate osteogenic gene expression in osteoblasts while upregulating adipogenic genes, effectively pushing bone cells toward a more fat-like phenotype (Clabaut et al., 2010). In fact, transcriptomic analyses of bone cells in aged individuals revealed that osteoblasts begin to express adipocyte-specific markers, suggesting a possible transdifferentiation or lineage switching in the bone marrow niche. This phenomenon is associated with changes in DNA methylation and is thought to contribute to the age-related decline in bone mass. The presence of adipogenic markers in osteoblasts from elderly subjects supports the idea that some bone-forming cells may convert to fat-storing cells over time, exacerbating osteoporosis risk (Clabaut et al., 2021). Compounding this problem, BM adipocytes in the aged niche secrete high levels of RANKL while expressing low levels of its decoy receptor osteoprotegerin (OPG). The resulting increase in osteoclast activity leads to greater bone resorption, thereby weakening bone structure. Collectively, these mechanisms help explain why most clinical studies associate high marrow fat with lower bone-mineral density and higher fragility-fracture risk in the elderly (Ho and Mendez-Ferrer, 2020).

### Inflammatory and metabolic alterations with BMAT expansion

BMAs are not merely passive fat storage cells; they actively secrete cytokines and adipokines that influence the bone marrow milieu and systemic metabolism. Aging is associated with chronic low-grade inflammation, and indeed pro-inflammatory cytokines increase in the bone marrow with age. This inflammatory shift can disrupt normal hematopoietic support and bone remodeling (Pritz et al., 2014). On the other hand, BMAs produce several anti-inflammatory and regulatory adipokines whose levels change with age, affecting both local and systemic physiology.

Adiponectin: Adiponectin is one of the most abundant anti-inflammatory adipokines in BMAT and plays beneficial roles in enhancing insulin sensitivity and glucose metabolism (Scherer et al., 1995; Pajvani et al., 2004). It also dampens inflammation by blocking NF-κB signaling, which reduces production of IL-6, IL-18, and TNFα. In aging bone marrow, adiponectin levels remain high (Chandrasekar et al., 2008; Yamaguchi et al., 2005); BMAT exhibits high expression of RANKL, regulated by C/EBPβ and C/EBPα, alongside the downregulation of OPG, a key inhibitor of osteoclast differentiation (Hu et al., 2021). While this may help counteract inflammation and metabolic dysregulation, paradoxically higher adiponectin in the elderly is also associated with reduced muscle mass and physical frailty (Karvonen-Gutierrez et al., 2016; Kizer et al., 2010). Thus, the role of adiponectin in ageing is complex: it confers metabolic benefits, but its elevated levels might reflect or contribute to age-related frailty.

SFRP5: Secreted frizzled-related protein 5 (SFRP5) is an adipokine that increases in aged BMAT. SFRP5 promotes adipogenesis and modulates Wnt signaling, but it also exerts anti-inflammatory effects by suppressing NF-κB activation and downregulating pro-inflammatory cytokines such as TNFα, IL-1β, and CCL2. Elevated SFRP5 in old age may thus help offset some inflammatory processes even as it encourages fat deposition (Song et al., 2013; Li et al., 2012; Xu et al., 2009; Fujimaki et al., 2001).

Omentin-1: Omentin-1, an adipokine associated with insulin sensitization, is found to increase with age and is linked to metabolic disturbances like obesity and diabetes. Higher omentin-1 in marrow fat may indicate a response to systemic metabolic stress in aging, although its direct effects on marrow cells remain to be fully understood (Watanabe et al., 2017; de Souza Batista et al., 2007).

C1q/TNF-Related Proteins (CTRPs): Several members of the CTRP family (e.g., CTRP1, CTRP3, CTRP9, CTRP12) are upregulated in aging adipose tissue and bone marrow. These adipokines generally enhance insulin sensitivity and glucose uptake, and some have specific anti-inflammatory actions (Kon et al., 2019; Liu et al., 2017). For instance, CTRP3 can inhibit TLR4-mediated inflammation, thereby reducing inflammatory cytokine production (Hofmann et al., 2011). In parallel, CTRP1, CTRP9, and CTRP12 improve insulin signaling and metabolic homeostasis (Peterson et al., 2012; Enomoto et al., 2011). While the increase of such adipokines in aged BMAT might mitigate chronic inflammation to a degree, their net impact on metabolism can be double-edged. Differential effects of various CTRPs and other adipokines on insulin signaling and lipid metabolism may contribute to paradoxical outcomes, potentially exacerbating conditions like obesity or diabetes in some contexts.

In summary, the accumulation of BMAT during aging has far-reaching consequences on the bone marrow ecosystem. Expanded BMAs in the elderly disrupt the equilibrium of MSC and HSC functions, altering both blood cell production and bone maintenance. Through a combination of altered cell-intrinsic pathways and changed secretory profiles (cytokines and adipokines), BMAs contribute to the immunosenescence and bone loss observed in older individuals. Importantly, the magnitude of these effects may vary depending on an individual’s health status. The molecular changes in the aged bone marrow niche, driven in part by BMAs, also have practical implications–for instance, they can influence immune reconstitution and hematopoietic recovery after bone marrow transplantation in elderly patients.

## BMAs in diseases

**FIGURE 2:**
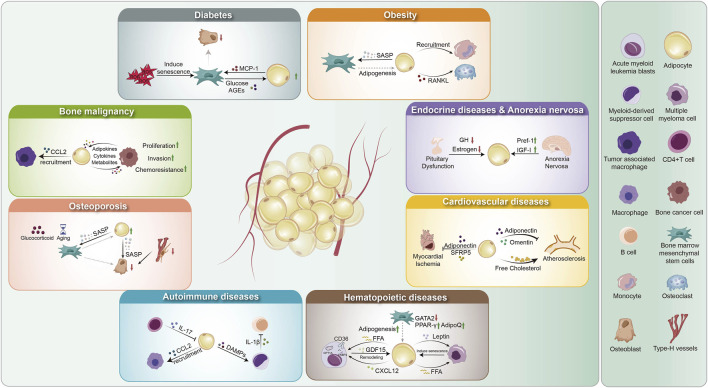
Overview of BMAs in disease pathology. (1) Hematopoietic diseases: BMAs enhance adipogenesis (regulated by GATA2, AdipoQ, PPAR-γ), remodel the microenvironment via GDF15 and CXCL12, and secrete FFAs and leptin, driving disease progression. (2) Bone malignancies: Adipocytes recruit tumor-associated macrophages via CCL2 and engage in bidirectional crosstalk with cancer cells through metabolites, adipokines, and cytokines, promoting proliferation, invasion, and chemoresistance. (3) Endocrine disorders and anorexia nervosa: Pituitary dysfunction reduces GH and estrogen, impairing BMA function. Anorexia nervosa upregulates Pref-1 and downregulates IGF-I, contributing to metabolic dysregulation. (4) Cardiovascular diseases: Myocardial ischemia decreases adiponectin and SFRP5, altering adipocyte function. BMAs also influence atherosclerosis through adipokine secretion (adiponectin, omentin) and cholesterol metabolism. (5) Diabetes: ROS-induced mesenchymal stem cell senescence disrupts adipogenesis and osteogenesis, while glucose and AGEs promote MCP-1 secretion, affecting bone homeostasis. (6) Obesity: BMAs drive mesenchymal stem cell senescence via SASP factors, promote adipogenesis, recruit monocytes, and secrete RANKL, enhancing osteoclast differentiation and bone resorption. (7) Autoimmune diseases: BMAs interact with immune cells by responding to IL-17 from CD4^+^ T cells, recruiting myeloid-derived suppressor cells via CCL2, and releasing DAMPs that modulate IL-1β production, contributing to immune dysregulation. GATA2: GATA-binding factor 2; AdipoQ: Adiponectin; PPAR: Peroxisome Proliferator-Activated Receptor; FFA: Free Fatty Acids; GDF15: Growth Differentiation Factor 15; CXCL12: C-X-C Motif Chemokine Ligand 12; GH: Growth Hormone; Pref-1: Preadipocyte Factor-1; IGF-1: Insulin-like Growth Factor 1; SFRP5: Secreted Frizzled-Related Protein 5; AGEs: Advanced Glycation End Products; MCP-1: Monocyte Chemoattractant Protein-1; SASP: Senescence-Associated Secretory Phenotype; RANKL: Receptor Activator of NF-kappa B Ligand; DAMPs: Damage-Associated Molecular Patterns; CCL2: C-C Motif Chemokine Ligand 2; MDSC: Myeloid-Derived Suppressor Cells.

Historically, most research on adipocytes in chronic and acute diseases has focused on WAT. However, growing evidence highlights the critical role of BMAT in maintaining bone marrow homeostasis and systemic metabolic regulation. Dysregulation of BMAT has been implicated in various pathological conditions, and conversely, systemic diseases can alter BMAT composition and function. Studies have explored the relationship between BMAT and diseases such as diabetes, obesity, cancer, cardiovascular disorders, hematologic disorders, and inflammatory conditions. This section of the review aims to provide a comprehensive understanding of the molecular and cellular mechanisms linking BMAT to these diseases (Figure 2).

### Diabetes and obesity

BMAT is increased in T2DM patients, who have increased blood glucose levels due to insulin resistance (Zhu et al., 2019; Sheu et al., 2017). Elevated glucose and reactive oxygen species (ROS) favour adipogenesis over osteogenesis by activating PPARγ and C/EBPα, thereby promoting marrow adipocyte differentiation (Rharass and Lucas, 2019). Elevated glucose levels induce excessive ROS production, contributing to insulin resistance and altered adipokine secretion (Rharass and Lucas, 2019). Additionally, adiponectin is downregulated in T2DM, impairing both BMAT function and glucose homeostasis, whereas leptin expression shows variable patterns depending on metabolic status (Rharass and Lucas, 2019). T2DM impairs bone marrow-derived PCs, which are critical for vascular stability and regeneration. Hyperglycemia and metabolic dysfunction reduce pericyte proliferation, viability, migration, and angiogenic capacity. These impairments are associated with the downregulation of key signaling pathways, including the AKT pathway, C-X-C motif chemokine ligand 2 (CXCL2), and angiopoietin-2, leading to compromised vascular integrity and impaired tissue repair (Mangialardi et al., 2019). The reduction of BM-derived PCs may influence BM adipogenesis. In type 1 diabetes mellitus (T1DM), the impact of BMAT appears to be less significant. While studies in mouse models have demonstrated a correlation between T1DM and BMAT, this association has not been observed in humans with T1DM (Carvalho et al., 2019; Botolin and McCabe, 2007). In a clinical study, thirty female patients with T1DM, matched for age and weight, showed no significant difference in vertebral BMAT compared to the control group (Abdalrahaman et al., 2015). Another study indicated that BMAT accumulation was associated with serum lipid levels rather than T1DM, highlighting the potential role of lipid homeostasis and adipogenic differentiation pathways in BMAT regulation (Slade et al., 2012).

While a reduction in adiponectin is linked to insulin resistance and T2DM, adipsin secreted by BMAT plays a crucial role in maintaining metabolic homeostasis. Adipsin enhances insulin secretion by pancreatic beta cells and protects them from apoptosis. A study demonstrated that adipsin preserves beta-cell function in diabetic mice, suggesting its therapeutic potential in diabetes management (Gomez-Banoy et al., 2019). This finding is promising, as most treatments for T2DM primarily target insulin resistance or enhance insulin production (Gomez-Banoy et al., 2019). Expanding therapeutic approaches to include factors related to BMAT may offer new strategies for diabetes management. Monocyte chemoattractant protein-1 (MCP-1), which is upregulated in the bone marrow of individuals with T2DM, promotes the differentiation of BM MSCs into the adipogenic lineage (Wan et al., 2023; Ferland-McCollough et al., 2018). Diabetic patients may receive insulin-sensitizing drugs such as TZDs, which target PPARγ, a key transcription factor regulating BMAT formation (Chen et al., 2017). In the skeletal system, DPP4 influences fracture healing by modulating osteoblast and osteoclast activity. RANKL, another BMAT-derived factor, promotes osteoclastogenesis and bone resorption, contributing to systemic inflammation and insulin resistance, which are implicated in T2DM pathogenesis (Kiechl et al., 2013; Ambrosi et al., 2017; Lamers et al., 2011). Intermittent PTH therapy indirectly inhibits RANKL signalling by activating PTH1R in BMAT, reducing marrow adiposity and limiting bone resorption, with potential benefits for insulin sensitivity (Fan et al., 2017). Furthermore, C1q/tumor necrosis factor-related proteins (CTRPs), particularly CTRP13, function as anti-inflammatory adipokines that enhance insulin sensitivity.

Furthermore, obesity is a complex metabolic disorder driven by genetic predisposition, environmental influences, and lifestyle factors (C et al., 2021). BMAT expands in obesity and is associated with osteoporosis and increased fracture risk, likely due to its influence on bone remodeling and metabolic regulation (Ambrosi et al., 2017; Styner et al., 2014). Deacetylation of PPARγ reduces its transcriptional activity, suppressing adipogenesis and improving insulin sensitivity and mitigating metabolic dysfunction in HFD-induced obese mice (Kraakman et al., 2018). As a result, BMAT increases, contributing to enhanced protection against TZDs-induced bone loss (Kraakman et al., 2018). PPARγ also upregulates FGF21, a metabolic regulator with potential therapeutic applications for obesity and diabetes. In turn, FGF21 positively regulates PPARγ activity and enhances the efficacy of TZDs-based anti-diabetic drugs (Dutchak et al., 2012). During obesity, BMAs secrete plasminogen activator inhibitor-1 (PAI-1), a key regulator of fibrinolysis and vascular function. High PAI-1 levels contribute to endothelial dysfunction and a pro-thrombotic state, linking BMAT expansion to cardiovascular risk factors. In the marrow, excess PAI-1 and similar factors can impair the microcirculation and nutrient supply, further stressing the bone niche.

Animal models have been instrumental in dissecting the relationship between obesity and BMAT. Studies utilizing HFD mouse models to investigate the relationship between BMAT and obesity further validate the effectiveness of this model. For example, in DKK1 knockout mice, HFD-induced BMAT expansion is reduced, suggesting that DKK1—normally upregulated in obesity—plays a role in BMAT regulation and metabolic dysfunction (Colditz et al., 2020). Additionally, HFD exposure leads to significant alterations in BMAT-associated gene expression, further highlighting the molecular adaptations of BMAT in response to obesity (Liu et al., 2013). For example, the expression of Krüppel-like factors 4 and 2 was upregulated, while coagulation factor II receptor-like 2 (F2RL2) and polo-like kinase 2 (PLK2) were downregulated. These gene expression changes suggest a shift in BMAT-associated regulatory pathways in response to metabolic alterations. In addition to HFD models, a lipodystrophic (fat-free) mouse model has been utilized to investigate the origins of BMAT in metabolic disorders such as diabetes (Zhang X. et al., 2021). This is a novel genetic congenital generalized lipodystrophy (CGL) model, in which CGL is a disorder characterized by complete loss of peripheral adipose tissue and is associated with diabetes, insulin resistance, hyperglyceridemia, and osteosclerosis (Zou et al., 2019; Fiorenza et al., 2011). They identified a secondary adipogenesis pathway that becomes activated with aging, driven by the recruitment of adiponectin-negative stromal progenitors. This shift results in BMAT with an enhanced capacity for lipid storage but a reduced ability to express key cytokines, including Cxcl12, adiponectin, Retn, and adipsin, which are crucial for maintaining bone and metabolic homeostasis (Zhang X. et al., 2021). Adiponectin is reduced in obesity and T2DM and increased in T1DM and during calorie restriction (Mancuso, 2016; Combs et al., 2003). The latter is linked to increased BMAT in obese and diabetic individuals (Miggitsch et al., 2019). For example, BMAT upregulates the expression of leptin, a pro-inflammatory adipokine that modulates immune responses, and promotes the recruitment of Ly6C^high^ monocytes, which contribute to systemic inflammation and insulin resistance during obesity (Boroumand et al., 2022; Mancuso and Bouchard, 2019). Leptin resistance is a hallmark of obesity. During obesity, B lymphopoiesis is suppressed due to a reduction in IL-7 levels within the BM (Adler et al., 2014). It can also disturb lipid draft/TGF-β, which maintains HSCs, in the BM of mice (Hermetet et al., 2019). Some studies have reported that HFD-induced obesity does not significantly alter the release of inflammatory cytokines from BMAT. For instance, levels of IL-1β and CCL2 remained unchanged (Hermetet et al., 2019; Nielsen et al., 2008). Additionally, another study found no evidence of HFD-induced BMAT inflammation but reported that non-obese mice exhibited higher mRNA expression of pro-inflammatory genes such as TNFα, IL-1β, and LCN2 (Tencerova et al., 2018). The findings in this section provide evidence that obesity and diabetes are associated with increased BMAT, potentially driven by dysregulated cellular and molecular mechanisms. However, further research is needed to elucidate the specific pathways linking BMAT expansion to metabolic dysfunction. Therefore, distinguishing the molecular signatures between these conditions is essential for understanding BMAT-related pathophysiology.

### Autoimmune diseases

Unlike obesity, which is characterized by low-grade chronic inflammation, high-grade inflammatory autoimmune diseases exhibit a stronger association with BMAT-related inflammatory factors. One such common inflammatory autoimmune disease is rheumatoid arthritis (RA) (Castro et al., 2017). BMAT-derived MSCs exhibit functional defects in RA patients. Specifically, these MSCs show a weaker ability to inhibit CD25 expression on autologous CD4^+^ and CD8^+^ T cells compared to allogeneic T cells (Kuca-Warnawin et al., 2020; Krajewska-Wlodarczyk et al., 2017). Another chronic inflammatory disease, spondyloarthritis, is associated with reduced BMAT levels in mouse models (Furesi et al., 2021). In the BM, neutrophils and IL-7-producing CD4^+^ T cells are increased, while erythroblasts are decreased. Among these immune cells and cytokines, IL-7 has been identified as a key regulator inhibiting adipogenesis in the BM(155). Beyond its effects on T cells, BMAT also inhibits B lymphopoiesis by promoting the differentiation of myeloid cells that produce IL-1β(17). This suppression of lymphopoiesis is associated with an increase in BMAT, IL-1β, and S100A9 expression by myeloid cells (Furesi et al., 2021). Additionally, BMAT upregulates several cytokines, including IL-10, IL-8, IL-6, TNFα, CCL2, CCRL2, and CXCL1 (Miggitsch et al., 2019). Nevertheless, BMAT produces anti-inflammatory adipokines (Mancuso, 2016; Mancuso and Bouchard, 2019). Among these, adiponectin activates AMPK in immune cells, potentially inhibiting inflammation by suppressing NF-κB signaling and reducing the production of pro-inflammatory cytokines such as TNFα, IL-6, and IL-8 (Mancuso, 2016). Conversely, adiponectin can promote COX-2 expression and enhance PGE2 synthesis in arthritic joints, thereby exacerbating inflammation and pain (Mancuso, 2016). Omentin inhibits TNFα-induced EC activation and, unlike adiponectin, suppresses COX-2 expression. SFRP5 reduces macrophage-derived TNFα, IL-1β, and CCL2/MCP-1 production (Guan et al., 2021). Given that BMAT influences both pro- and anti-inflammatory adipokines, it plays a crucial role in modulating immune and inflammatory responses associated with these signaling molecules. The studies discussed in this paragraph, along with findings from other sections, highlight the significant role of BMAT in both promoting and suppressing inflammatory cytokines.

### Endocrine diseases and anorexia nervosa

BMAT accumulation is tightly regulated by endocrine signaling, particularly through GH, estrogens, and glucocorticoids. Pituitary dysfunction disrupts the secretion of these hormones, leading to alterations in BMAT homeostasis. In hypophysectomized rats, the loss of pituitary-derived GH results in increased BMAT, likely due to impaired lipolysis and adipocyte differentiation. These findings underscore the essential role of GH-mediated molecular pathways in maintaining BMAT balance. Other endocrine factors, including thyroxine, cortisone and IGF-1, failed to reverse BMAT expansion (Menagh et al., 2010). Conversely, BMAT itself may modulate endocrine homeostasis through molecular signaling pathways. For instance, during caloric restriction, increased BMAT accumulation correlates with elevated glucocorticoid levels (Piotrowska and Tarnowski, 2021; Polineni et al., 2020). This suggests a bidirectional regulatory mechanism between BMAT and systemic endocrine responses.

Endocrine factors may also influence BMAT dynamics in diseases such as anorexia nervosa. In premenopausal women with anorexia nervosa, BMAT decreases following transdermal estrogen treatment. This reduction is associated with an increase in red blood cell count and hematocrit levels, suggesting a potential link between estrogen signaling, BMAT regulation, and hematopoiesis (Polineni et al., 2020). Previous studies have demonstrated that BMAT is increased in patients with anorexia nervosa (Doucette et al., 2015). This increase may be partially explained by elevated levels of DLK1 observed in women with anorexia (Piotrowska and Tarnowski, 2021; Devlin and Rosen, 2015). Supporting this, DLK1 levels decline during anorexia treatment, coinciding with reductions in BMAT and bone loss (Fazeli et al., 2012). Moreover, unlike obesity and T2DM, anorexia nervosa is associated with elevated adiponectin levels (Cawthorn et al., 2014). As BMAT is an endocrine organ, further research is needed to elucidate the molecular mechanisms linking BMAT to endocrine disorders and metabolic adaptations in anorexia nervosa.

### Cardiovascular diseases

Unlike in some other disorders, BMAT-derived adipokines may exert a protective role in cardiovascular disease. Additionally, given that adiponectin plays a key role in metabolic regulation, it may also influence cardiovascular pathology, provides a possibility for it to affect cardiovascular disorders. For example, adiponectin, similar to SFRP5, has a protective role in myocardial ischemia by reducing cellular infiltration and modulating inflammatory responses (Nakamura et al., 2016; Shibata et al., 2005). Additionally, adiponectin may protect against aortic aneurysm by attenuating vascular inflammation (Yoshida et al., 2014). Omentin, another adipokine, plays a protective role in vascular health. It mitigates vascular inflammation associated with atherosclerosis by downregulating the expression of intracellular adhesion molecule-1 (ICAM-1) and vascular cell adhesion molecule-1 (VCAM-1). Additionally, cholesterol is another crucial factor in the development and progression of atherosclerosis. BMAT increases during cholesterol metabolism, characterized by elevated free cholesterol levels and reduced expression of proteins associated with lipolysis. This alteration suggests that BMAT may contribute to the development of atherosclerosis (Attané et al., 2019). In contrast to other adipokines, adipsin does not influence atherosclerosis development in LDL receptor-knockout mice. However, its effects in humans may differ (Liu L. et al., 2021). The impact of BMAT on cardiovascular disorders varies among adipokines, highlighting the need for further research to establish clearer correlations.

### Osteoporosis

Osteoporosis is a skeletal and metabolic disorder characterized by reduced bone mass and disruption of the bone microenvironment, primarily driven by an imbalance between osteoblast-mediated bone formation and osteoclast-driven bone resorption (Langdahl, 2021). One of the most common forms is glucocorticoid-induced osteoporosis (GIOP) (Kusumbe et al., 2016). Given that BM adipocytes and osteoblasts share a common progenitor lineage, BMAT is implicated in osteoporosis. Recent studies have shown that osteoporosis is associated with a shift in BMAT lipid composition, characterized by a reduction in unsaturated lipids and an increase in saturated lipids (Bao et al., 2023).

Overall, it remains unclear whether increased BMAT directly contributes to bone loss, occurs as a consequence of bone loss (Ambrosi et al., 2017). Some studies have indicated that an increase in BMAT is associated with reduced BMD in both aged humans and animals (Schwartz et al., 2013; Duque et al., 2009). Other studies have reported that in mice, BMAT expansion occurs later in life, around 2 months after the onset of bone loss, suggesting a temporal relationship between these processes (Glatt et al., 2007; Lazarenko et al., 2007). Moreover, a study reported increased bone loss without changes in BMAT in mice deficient in 11β-hydroxysteroid dehydrogenase type 1 (11β-HSD1) (Justesen et al., 2004). Elevated BMAT has been observed in postmenopausal osteoporosis, further highlighting its potential role in bone metabolism (Li J. et al., 2020).

Furthermore, osteoporosis treatment strategies can target factors that promote BMAT expansion and suppress bone formation. For example, sclerostin, a key inhibitor of the Wnt signaling pathway, can be neutralized by the humanized monoclonal antibody Romosozumab, which enhances bone formation and reduces bone resorption (McClung et al., 2014). Similarly, antibodies targeting DKK1, another Wnt pathway inhibitor, have been shown to increase bone mass and facilitate bone fracture repair (Florio et al., 2016). The treatment of osteoporosis has its disadvantages too. For example, glucocorticoid exposure induces GIOP can have side effects such as bone loss and fracture, which occurs in about 30%–50% of GIOP patients (Florio et al., 2016). Another potential therapeutic approach involves promoting vascular formation, as impaired angiogenesis has been linked to osteoporosis and reduced bone regeneration. Therefore, desferrioxamine, when administered to osteoporotic mice, has been shown to increase H-type vessel formation and prevent bone loss (Wang et al., 2017b). Another key factor is βII-spectrin (SPTBN1), which enhances the expression of VEGF, a key regulator of angiogenesis, thereby promoting both bone formation and blood vessel development in osteoporotic conditions (Xu et al., 2021). Lifelong osteoporosis treatment can be costly, particularly when the condition arises as a consequence of irradiation or chemotherapy for solid or hematopoietic tumor treatment (Devlin and Rosen, 2015). A promising approach involves altering the irradiated BMAT lineage to favor osteogenesis over adipogenesis, potentially improving bone regeneration (Devlin and Rosen, 2015). However, the development of more cost-effective and safer therapeutic strategies with fewer side effects remains crucial. Given the significant role of BMAT in the onset and progression of postmenopausal osteoporosis, targeting BMAT-associated factors presents a promising avenue for future osteoporosis treatments.

### Hematopoietic diseases

BMAT plays a crucial role in regulating the hematopoietic microenvironment. Dysregulation of BMAT may contribute to the progression of hematopoietic malignancies, such as acute myeloid leukemia (AML) and multiple myeloma (MM). Notably, an increased presence of small BM adipocytes has been observed in AML patients, which may serve as a potential biomarker for poor prognosis in AML (Lu et al., 2018a; Feldman et al., 2006). BMAT has been found to secrete FFAs, which are transported to AML cells following BMAT-induced lipolysis via FABP4 (Shafat et al., 2017; Lu et al., 2018a; Feldman et al., 2006; Cuminetti and Arranz, 2019). Various therapeutic strategies have been explored to target AML, including the inhibition of FABP4 and carnitine palmitoyltransferase 1a (CPT1a), an enzyme responsible for lipid transport into mitochondria for β-oxidation. Blocking FABP4 and CPT1a disrupts AML cell lipid metabolism, ultimately leading to AML cell death (R et al., 2015). BMAT is implicated in leukemia progression through multiple signaling pathways. These cells, in turn, express leptin receptors, which are highly upregulated in primary acute promyelocytic leukemia (APL) (Tabe et al., 2004; Konopleva et al., 1999). This cell-cell interaction facilitates APL progression by promoting leukemic cell survival and proliferation. Additionally, BMAT secretes CXCL12, thereby supporting their migration, survival, and drug resistance (Cho et al., 2017). Therefore, inhibiting CXCL12/CXCR4 is a potential therapy way for AML.

Just as BMAT influences AML progression, AML cells can also impact adipogenesis. AML cells secrete growth differentiation factor 15 (GDF15), which reduces BMAT volume and adipocyte size (Lu et al., 2018b). This disruption contributes to impaired myelo-erythroid maturation, further compromising hematopoietic homeostasis (Wang et al., 2018). While targeting CXCL12 and FABP4 has emerged as a promising therapeutic strategy against AML, some treatments have limitations concerning BMAT. For instance, dexamethasone resistance can develop in lymphocytic leukemia, potentially reducing treatment efficacy and complicating disease management (Tung et al., 2013).

Moreover, BMAT plays a key role in promoting the growth and survival of MM cells, a malignancy of plasma cells, a subset of white blood cells. BMAT-derived leptin enhances MM cell proliferation; however, this effect diminishes with treatment (Fotiou and Katodritou, 2025). Interestingly, this process can be hindered by drug resistance. BMAT contributes to MM drug resistance by creating a supportive microenvironment that enhances tumor cell survival, ultimately reducing treatment efficacy and facilitating disease progression (Fotiou and Katodritou, 2025). Leptin upregulate autophagic proteins in MM cells, contributing to chemotherapy resistance (Liu et al., 2015). Resistance can be mitigated by alternative therapeutic strategies, such as inhibiting fatty acid-binding proteins (FABPs) in MM cells. Targeting FABPs disrupts lipid metabolism, thereby reducing MM cell proliferation and enhancing treatment responsiveness (Fotiou and Katodritou, 2025). Other BMAT-derived factors, such as MCP-1/CCL2 and stromal cell-derived factor-1α, function as chemotactic agents for multiple myeloma cells (Fairfield et al., 2021). Similar to its role in leukemia, dexamethasone is also utilized in the treatment of multiple myeloma. Furthermore, while BMAT influences MM, MM cells, in turn, modulate BMAT adipogenesis (Fairfield et al., 2021; Fairfield et al., 2020).

Aplastic anemia, a hematopoietic disorder characterized by bone marrow failure and reduced hematopoiesis, is associated with increased BMAT (Tripathy et al., 2014). At the cellular level, bone marrow stromal cells (BMSCs) in aplastic anemia exhibit a skewed differentiation towards adipogenesis over osteogenesis, contributing to impaired hematopoietic support. Molecularly, this shift is driven by dysregulated signaling pathways. GATA2, a transcription factor critical for HSCs maintenance, functions as a BMAT suppressor by inhibiting PPARγ-mediated adipogenesis. In aplastic anemia, reduced GATA2 expression leads to dysregulated MSCs differentiation, favoring adipocyte formation over osteoblast lineage commitment. This reduction enhances adipogenic pathways, contributing to excessive BMAT accumulation (Xu et al., 2009; Fujimaki et al., 2001). Therefore, therapeutic strategies aimed at enhancing GATA2 expression may help mitigate BMAT expansion in these patients.

Collectively, these studies highlight the critical role of BMAT-derived factors in influencing various diseases, while also demonstrating how these diseases, in turn, modulate BMAT composition. The most affected conditions are those associated with disruptions in the bone marrow microenvironment. However, due to the heterogeneity of BMAT in metabolic and hematopoietic disorders, further research is needed to establish a more precise understanding of the correlations between BMAT, its secreted factors, and disease pathophysiology.

### Bone malignancy

The preceding discussion underscores the ability of BMAT to interact with tumor cells through its role as an endocrine regulator of the skeletal and bone marrow microenvironments (Cawthorn et al., 2014; Morris and Edwards, 2016). Maintaining homeostasis in the bone microenvironment requires a tightly regulated balance between angiogenesis, adipogenesis, and osteogenesis. Dysregulation of these pathways can lead to pathological remodeling, promoting tumor cell survival and disease progression. This microenvironment facilitates the metastasis of breast cancer, prostate cancer, and MM, as well as the progression of primary bone cancers (Guerra et al., 2018). Additionally, the bone marrow vascular niches play a crucial role in supporting tumor growth by enabling extensive crosstalk between cancer cells and various cellular components within the bone marrow (Godavarthy et al., 2020).

The structural and functional characteristics of bone marrow vasculature, including its large-diameter sinusoidal vessels and sluggish blood flow, contribute to cancer therapy resistance by limiting drug perfusion and immune cell infiltration. Although primary bone cancer is rare, accounting for only 1% of all cancers, it is one of the most painful malignancies. Its morbidity is more prevalent among children and young adults compared to the elderly (Marengo et al., 2011). Bone cancer encompasses various types, including osteosarcoma, Ewing sarcoma, chondrosarcoma, and chordoma, with osteosarcoma being the most common primary malignant bone tumor (Obmolova et al., 2012). However, bone malignancies can also arise from metastatic spread of other cancers, particularly hematopoietic malignancies originating in the bone marrow, such as lymphoma, leukemia, or MM.

Most studies exploring the link between bone cancer and adipocytes have focused on WAT rather than BMAT, partly due to the technical challenges of isolating and culturing BMAT. Consequently, BM-MSCs are commonly used as a model to induce BMAT differentiation. BMAT is known to have some differences in gene expression of lipid synthesis, signaling pathways, and proteins compared to other types of adipocytes, but also a lot of similarities (Ferguson and Turner, 2018). For example, PPARγ signaling is upregulated in BMAT, similar to WAT (Beekman et al., 2019a). This suggests that BMAT may influence bone cancer progression through some mechanisms shared with WAT, such as modulation of tumor growth, metastasis, and drug resistance, while also exerting unique marrow-specific effects.

Bone cancer secretes various factors and cytokines that influence the surrounding microenvironment, modulating the activity of osteoblasts, osteoclasts, and BMAT. In response, BMAT may contribute to the progression of bone malignancies. BMAT releases several adipokines, which have pro-tumorigenic effects by promoting cancer cell proliferation, survival, and metastasis. For example, IL-6 promotes tumor cell survival and facilitates metastasis (Reagan et al., 2021; Walter et al., 2009). Leptin enhances cancer cell colonization, autophagy, and chemoresistance by activating the JAK2/STAT3 and RAS signaling pathways, creating a feedback loop that further supports tumor survival (Yue et al., 2016; Dirat et al., 2011). Additionally, BMAT-derived cyclooxygenase-2 (COX-2) and PGE2 contribute to tumor progression by inducing angiogenesis, facilitating immune evasion, and promoting tumor-associated bone degradation. BMAT increases the release of FFAs, which activate the PI3K/AKT and NF-κB signaling pathways, driving tumor proliferation and upregulating COX-2 expression, leading to increased PGE2 synthesis (Cha and Koo, 2019; Ye et al., 2016). Additionally, BMAT secretes CCL2, which binds to its receptor CCR2 on monocytes and ECs, stimulating angiogenesis and tumor-associated macrophage recruitment (Luo et al., 2018). BMAT also secretes RANKL, which is known to support osteosarcoma and chondrosarcoma (Beekman et al., 2019b; Herroon et al., 2013; Evola et al., 2017; Marley et al., 2015). Furthermore, BMAT enhances tumor growth in bones through the FABP4 pathway, which also promotes adipogenesis in a reciprocal manner (Arendt et al., 2013). High expression of FABP4, along with CD36 and perilipin 2, facilitates lipid transfer from BMAT to tumor cells. BMAT-derived FFAs are transported via CD36 into the cytosol and subsequently shuttled by FABP4 to the mitochondria, where they undergo β-oxidation. This process is regulated by carnitine palmitoyltransferase-1A (CPT1A), driving ATP production to fuel tumor cell metabolism and proliferation (Shafat et al., 2017; Pascual et al., 2017; Tirode et al., 2007). Besides, FFAs can be transported to the nucleus by FABP4, where they act as ligands for the nuclear receptor PPARγ. This activation leads to the upregulation of anti-apoptotic factors such as Bcl-2, promoting tumor cell survival and resistance to apoptosis (Herroon et al., 2013). In turn, tumor cells also promote adipogenesis by stimulating LEPR^+^/Sca-1^+^ BMSCs to differentiate into adipocytes. A notable example of a bone tumor associated with this mechanism is Ewing sarcoma, the second most common primary bone cancer. In Ewing sarcoma, suppression of the FLI1 gene leads to a moderate increase in FABP4 expression, potentially influencing tumor metabolism and microenvironment interactions (Takeshita et al., 2014).

Given the tumor-supporting role of BMAT in bone cancer, it provides a potential therapeutic target for treatment. The differentiation preference of BMSCs in the bone cancer microenvironment can be modulated by targeting key regulators such as PPARγ, glucocorticoids, and TZDs. Additionally, targeting protein kinase C has been shown to enhance osteogenesis while exhibiting anti-tumor effects, making it a promising strategy for bone cancer therapy (David et al., 2011). Another therapeutic approach involves targeting lipid metabolism, which plays a crucial role in supporting cancer cell survival. Inhibiting fatty acid synthesis and uptake, as well as key enzymes involved in lipid metabolism—such as fatty acid synthase, acetyl-CoA carboxylase, and ATP-citrate lyase—can impair tumor cell survival and proliferation by disrupting energy production and membrane biosynthesis. In contrary, certain cancer therapies, such as dexamethasone and irradiation, have been shown to increase BMAT levels (Hess et al., 2006). Fasudil, an inhibitor of Rho-associated coiled-coil containing protein kinase (ROCK), promotes terminal adipocyte differentiation while suppressing *in vivo* tumorigenesis in chemoresistant osteosarcoma cells (Tabe et al., 2017). Fasudil exerts its effects by inhibiting megakaryoblastic leukemia 1 (MKL1), thereby preventing MKL1-mediated suppression of PPARγ, which leads to enhanced adipogenesis in osteosarcoma cells (Tabe et al., 2017; Rosenwald et al., 2017). Other drugs, such as TZDs—including rosiglitazone, troglitazone, and pioglitazone—modulate PPARγ activity, enhance adipogenesis, and exhibit anti-tumor effects (Takahashi et al., 2019; Yousefnia et al., 2018). However, despite their therapeutic potential, TZDs have been associated with adverse effects, including weight gain and bone loss, which may limit their clinical application (Wagner et al., 2010).

Overall, despite the recognized importance of BMAT in the tumor microenvironment, relatively few studies have specifically examined its correlation with bone cancer. Most existing research focuses either on other adipocyte types, such as WAT, or on different cancers, including MM, leukemia, and breast cancer. Although current studies highlight the potential role of BMAT in bone cancer progression and treatment, further research is needed to elucidate the specific contributions of BMAT-derived factors in bone cancer pathophysiology.

### Lymphatic–adipocyte crosstalk in the bone marrow microenvironment

Recent identification of lymphatic endothelial cells (LECs) in bone has opened new avenues for understanding their interactions with bone marrow adipocytes and adipocyte progenitors (Biswas et al., 2023; Liu et al., 2025b). LECs secrete molecular mediators that modulate immune cell trafficking and may influence adipogenic niches by shaping the local cytokine and chemokine environment. Conversely, adipocytes and their precursors produce adipokines—including leptin and adiponectin—as well as pro-inflammatory cytokines like TNF-α and IL-6, which can regulate lymphangiogenic signaling and lymphatic remodeling (Li et al., 2025). In the bone marrow, this bidirectional crosstalk may impact osteoimmune regulation, lipid metabolism, and skeletal integrity. While such interactions are well-characterized in peripheral adipose depots, their specific roles within the bone microenvironment remain largely uncharacterized. Further mechanistic studies are needed to delineate how LECs and adipogenic cells co-regulate bone physiology under conditions such as ageing, obesity, and inflammation.

## Conclusion and future perspective

In conclusion, recent studies have provided greater insights into the physiological and pathophysiological functions of BMAT. The bone marrow is a critical organ involved in metabolic, hematopoietic, immune, skeletal, and nervous system regulation. Given that BMAT constitutes approximately 70% of the bone marrow’s volume, it plays a significant role in maintaining systemic homeostasis. However, despite these advancements, research on BMAT has also raised new questions, highlighting the need for further investigation into its precise functions and interactions within the bone marrow microenvironment. These questions primarily pertain to the role of BMAT in disease pathology rather than its contribution to systemic homeostasis. Notably, species-specific differences in BMAT function have been observed, with murine BMAT secreting distinct factors compared to human BMAT, suggesting variations in adipokine signaling and metabolic regulation. For example, the correlation between T1DM and BMAT has been established in murine models but remains unclear in humans, possibly due to differences in insulin signaling, inflammatory cytokine profiles, or adipocyte differentiation pathways. Given the challenges of directly studying human BMAT, future research should leverage advanced humanized models, such as organ-on-a-chip systems and BM-MSC differentiation platforms, to better elucidate the cellular and molecular mechanisms governing BMAT function in disease progression. Further research is needed to characterize BMAT-specific factors, as many cytokines and adipokines secreted by BMAT, such as leptin, adiponectin, and IL-6, are also produced by other adipose tissue types, complicating their functional distinction. Additionally, aging, chronic diseases, and metabolic dysregulation alter the crosstalk between BMAT, vascular niches, and hematopoietic or MSCs-derived populations in the bone marrow microenvironment. These disruptions influence key signaling pathways, such as PI3K/AKT, NF-κB, and CXCL12/CXCR4, leading to impaired hematopoiesis, immune dysfunction, and enhanced tumor progression. Distinct molecular differences between BMAT and other adipose tissue types highlight the need for further investigation into BMAT-specific factors and their role in disease progression. Future research should focus on the molecular crosstalk between BMAT and vascular niches, particularly the influence of BMAT-derived cytokines (e.g., IL-6, CXCL12) and adipokines (e.g., leptin, adiponectin) on endothelial function, hematopoiesis, and tumor-stroma interactions. Understanding these interactions at a cellular and molecular level will provide new insights into BMAT’s contribution to bone marrow-associated disorders and potential therapeutic targets.

Currently, the assessment of human BMAT primarily relies on magnetic resonance spectroscopy imaging techniques and histological analysis. However, these methods face several key limitations: (1) Spatial resolution constraints: 1H-Magnetic Resonance Spectroscopy, the gold standard for BMAT quantification, is restricted to single-voxel analysis, inadequately capturing heterogeneous BMAT distribution in metabolically active regions (Singhal and Bredella, 2019). (2) Protocol variability: Despite standardization efforts by the bone marrow adiposity society, inconsistencies in water-fat imaging parameters hinder cross-study comparability (Jarraya and Bredella, 2021). (3) Reductionist approaches: Most studies fail to integrate BMAT secretory profiles (e.g., osteocalcin, adiponectin) with systemic biomarkers, limiting insights into the metabolic-bone-vascular axis.

As a result, BMAT quantification remains challenging in clinical settings, highlighting the urgent need for the development and validation of novel non-invasive imaging modalities that can accurately assess BMAT volume and distribution in human patients. Advancements in imaging technologies may facilitate research on how BMAT correlates with key physiological and pathological parameters, including inflammation, insulin sensitivity, bone density, endothelial function, arterial stiffness, and systemic atherosclerosis. Given the observed increase in BMAT in aging-related diseases such as obesity and diabetes, investigating the relationship between BMAT quantification and inflammatory biomarkers in these patients would be particularly valuable. Future studies focusing on these associations could deepen our understanding of the role of BMAT in both health and disease.

## Glossary

AML: Acute myeloid leukemia
AR: Adrenergic receptor
APL: Acute promyelocytic leukemia
BAT: Brown adipose tissue
BM: Bone marrow
BMA: Bone marrow adipocyte
BMAL1: Brain and muscle ARNT-like protein 1
BMAT: Bone marrow adipose tissue
BMD: Bone mineral density
BMSC: Bone marrow stromal cell
CAR: CXCL12-abundant reticular
CGL: Congenital generalized lipodystrophy
CPT1a: Carnitine palmitoyltransferase 1a
DKK1: Dickkopf-related protein 1
DPP4: Dipeptidyl peptidase-4
EC: Endothelial cell
HSC: Hematopoietic stem cell
FFAs: Free fatty acids
FABP4: Fatty acid-binding protein 4
IGF-1: Insulin-like growth factor 1
LEPR: Leptin receptor
MAT: Marrow adipose tissue
MCP: Monocyte chemoattractant protein
MM: Multiple myeloma
MSC: Mesenchymal stem cell
OPG: Osteoprotegerin
OPN: Osteopontin
PPAR: Peroxisome proliferator-activated receptor
PTH: Parathyroid hormone
RA: Rheumatoid arthritis
RANKL: Receptor activator of NF-kappa B ligand
SCF: Stem cell factor
TNF: Tumor necrosis factor
VEGF: Vascular endothelial growth factor
WAT: White adipose tissue
