# Supplementary material for: Bone marrow adipocytes: key players in vascular niches, aging, and disease
Source: Front Cell Dev Biol. 2025 Aug 7;13:1633801. doi: 10.3389/fcell.2025.1633801 (PMC12367753; doi:10.3389/fcell.2025.1633801)
Supplement: Supplementary file 1 [file Table1.docx]

**Table S1.** Summary of characterizations of the different types of adipocytes BMAT, WAT, BAT, BeAT, and PAT.

| **Adipocyte type** | **BMAT** | **WAT** | **BAT** | **BeAT** | **PAT** |
| --- | --- | --- | --- | --- | --- |
| Anatomical location | cBMAT: Distal long bones.  rBMAT: Proximal regions of long bones and spinal vertebrae | Subcutaneous (abdominal, femoral-gluteal) Visceral (mesenteric, omental, retroperitoneal, perigonadal) regions | Supraclavicular,  Periadrenal,  Paravertebral,  Cervical,  Axillary,  Paraspinal regions | Supraclavicular regions | Mammary gland |
| UCP-1 level | Low expressed | Low expressed | High expressed | High expressed | Low expressed |
| Mitochondrial number | Medium | Low | High | Medium | Not known |
| Vascularization | Medium | Low | High | High | Not known |
| Functions | Hematopoiesis,  Bone remodeling,  Cholesterol metabolism,  Adipokine-immune regulation | Energy and fat storage,  Endocrine organ regulating hunger,  Glucose homeostasis | Non-shivering thermogenesis mediated by UCP1 | Thermogenesis is UCP1-independent and is mainly regulated through Ca2+ and creatine cycling | Induces milk secretion during lactation |

BMAT, bone marrow adipose tissue; WAT, white adipose tissue; BAT, brown adipose tissue; BeAT, beige adipose tissue; PAT, pink adipose tissue; cBMAT, constitutive BMAT; rBMAT, regulated BMAT; UCP1, uncoupling protein 1.
