# Supplementary material for: Bone marrow adipocytes: key players in vascular niches, aging, and disease
Source: Front Cell Dev Biol. 2025 Aug 7;13:1633801. doi: 10.3389/fcell.2025.1633801 (PMC12367753; doi:10.3389/fcell.2025.1633801)
Supplement: Supplementary file 2 [file DataSheet1.docx]

**S1. Differences between bone marrow- and other adipocytes**

Extensive literature characterizes BMAT as a dynamic endocrine organ that regulates systemic energy homeostasis through the secretion of adipokines and metabolic regulators(Zinngrebe, Debatin, & Fischer-Posovszky, 2020). The first adipocytes were described in 1551, and these cells are now identified as brown adipocytes(Trayhurn, 2022) . Adipocytes are now classified based on their stem cell origin, molecular signatures, physiological and metabolic functions, and specific protein biomarkers(Bukowska et al., 2018). Adipocytes are primarily distributed in subcutaneous adipose tissue (SAT), located beneath the skin, which constitutes approximately 80% of total body fat, while the remaining 20% is found in visceral adipose tissue (VAT), surrounding internal organs such as the mesentery and kidneys(Neamat-Allah et al., 2014; Tsiloulis & Watt, 2015). To date, five distinct types of adipose tissue have been identified, each differentiating from adipose progenitor cells (APCs): white (WAT), brown (BAT), beige (BeAT), pink adipose tissue (PAT), and BMAT, also referred to as yellow adipocytes. Among these, WAT and BAT are the two primary subtypes, exhibiting distinct molecular signatures and functional properties(Cheng et al., 2021). Lineage-tracing studies in mice have provided critical insights into the developmental origins of mature adipocytes. Using the Cre/loxP recombination system, Myf5-Cre mice crossed with R26R reporter mice demonstrated that brown adipocytes and skeletal muscle cells originate from Myf5⁺ progenitors, whereas white adipocytes derive from Myf5⁻ progenitors. This finding suggests that brown adipocytes share a common developmental lineage with skeletal muscle cells, distinguishing them from white adipocytes at the molecular level(Cristancho & Lazar, 2011). In addition to lineage-tracing studies, fibroblasts residing in the adventitia of blood vessels contribute to the differentiation of BAT, BeAT, and WAT by expressing platelet-derived growth factor receptors PDGFR-β and PDGFR-α(Cattaneo et al., 2020). The vascular niche plays a crucial role in regulating adipogenesis across various adipose depots. APCs contribute to this process by secreting a range of paracrine factors, including VEGF, ANGPTL4, fibroblast growth factor 2 (FGF-2), and matrix metalloproteinases (MMPs), which collectively influence adipocyte differentiation, extracellular matrix remodeling, and angiogenesis. The secretion of these factors is partially regulated by PPARγ, a master regulator of adipogenesis. Additionally, APCs exhibit a distinct cell surface marker profile characterized by CD45⁻ CD31⁻ CD34⁺ CD29⁺ SCA1⁺ CD24⁺/⁻, which enables their identification and functional characterization within the adipose microenvironment(Sebo & Rodeheffer, 2019). These APCs originate from a multipotent precursor characterized by the surface marker profile CD45⁻ CD31⁻ SCA1⁺ CD24⁺, which also gives rise to osteogenic precursors. Recent studies have identified three distinct APC subpopulations based on CD34 expression: CD34-high, CD34-low, and CD34-negative. These subpopulations exhibit differences in metabolic activity, endocrine function, and differentiation potential. Furthermore, their distribution varies in individuals with type 2 diabetes mellitus (T2DM), suggesting a role in adipose tissue remodeling and metabolic dysregulation associated with insulin resistance(Raajendiran et al., 2019).

Beyond APCs differentiation into specific adipocyte lineages, there is dynamic interconversion between adipocyte subtypes. WAT can undergo 'beiging,' a process in which white adipocytes acquire thermogenic properties similar to BeAT. Also, beige adipocytes exhibit plasticity and can revert to WAT during weight gain and excess lipid accumulation, a process known as 'whitening.' Conversely, WAT can undergo 'browning,' a phenomenon in which white adipocytes acquire thermogenic properties similar to BAT. Browning is enhanced by physiological and metabolic stimuli such as weight loss, cachexia, cold exposure, exercise, and bariatric surgery. Additionally, transcriptional regulators PRDM16 and SMAD3 promote brown adipocyte-like characteristics, while pharmacological and dietary interventions, including PPAR agonists, thiazolidinediones (TZDs), conjugated linoleic acid, and non-caffeinated green tea extract, further stimulate the thermogenic gene program. These molecular mechanisms highlight the dynamic plasticity of adipose tissue in response to metabolic and environmental cues(Chait & den Hartigh, 2020; Correa, Heyn, & Magalhaes, 2019; Cristancho & Lazar, 2011; Giralt & Villarroya, 2013; Sanchez-Gurmaches & Guertin, 2014). Both WAT and BAT can transdifferentiate into PAT during pregnancy and lactation. This process is regulated by the upregulation of the transcription factor secreted phosphoprotein 1 (SPP1) and a concurrent reduction in PPARγ expression, leading to the differentiation of adipocytes into milk-secreting mammary gland-associated cells(Prokesch et al., 2014; Wang et al., 2018). Following lactation, PAT can revert to either WAT or BAT, highlighting the plasticity of adipose tissue in response to reproductive and metabolic demands(Pant, Firmal, Shah, Alam, & Chattopadhyay, 2020).

To better understand the differences and similarities among various adipocyte subtypes, their signaling pathways and physiological roles will be explored in detail in the following sections.

**BAT**

Although BAT constitutes only 1–2% of total body fat, it is among the most highly vascularized adipose depots, enabling rapid oxygen and nutrient supply to sustain thermogenesis(Kahn, Wang, & Lee, 2019; Shimizu et al., 2014). BAT is exclusively present in mammals and is characterized by a dense mitochondrial network expressing uncoupling protein 1 (UCP1), which facilitates proton leakage across the inner mitochondrial membrane, dissipating energy as heat(Craft & Scheller, 2017; Gesta, Tseng, & Kahn, 2007; Ikeda, Maretich, & Kajimura, 2018). Using ¹⁸F-FDG PET/CT imaging, BAT has been detected in specific anatomical regions in adults, including the supraclavicular, cervical, perirenal, and paravertebral areas. However, adult BAT is molecularly and functionally distinct from neonatal BAT, exhibiting lower thermogenic activity and reduced UCP1 expression due to age-related metabolic adaptations (Table S1)(D. C. Berry, Stenesen, Zeve, & Graff, 2013; Cheng et al., 2021; Cypess et al., 2009; Ikeda et al., 2018; Kahn et al., 2019; Sanchez-Gurmaches & Guertin, 2014; Virtanen et al., 2009; Wu et al., 2012). Compared to WAT, BAT adipocytes are smaller in size (15–60 μm) and exhibit a distinct phenotype characterized by numerous mitochondria with densely packed cristae, abundant cytoplasm, and multiple small lipid droplets(Carrageta, Oliveira, Monteiro, & Alves, 2020; Reddy, Tan, Barber, & Randeva, 2014; Saely, Geiger, & Drexel, 2012). The characteristic brown coloration of BAT is attributed to the high mitochondrial density and elevated cytochrome c content, which facilitates oxidative phosphorylation and thermogenesis(Cinti, 2009; Saely et al., 2012; Zinngrebe et al., 2020).

BAT is primarily responsible for heat production. This thermogenic function explains the high mitochondrial density and extensive capillary network surrounding BAT, which ensures efficient oxygen and nutrient delivery to support oxidative phosphorylation and lipid metabolism (Table S1)(Saely et al., 2012). During this process, UCP1, located in the inner mitochondrial membrane, disrupts the coupling between oxidative phosphorylation and ATP synthase activity, allowing protons to leak across the membrane. This uncoupling dissipates the electrochemical gradient as heat rather than driving ATP production, thereby enhancing fatty acid oxidation (FAO) to sustain thermogenesis(Bartelt & Heeren, 2014; Chouchani, Kazak, & Spiegelman, 2019; Lidell, Betz, & Enerback, 2014). Thermogenesis has been shown to enhance glucose uptake, improve insulin sensitivity, regulate glycolipid metabolism, mitigate lipid accumulation, and maintain body temperature homeostasis. Consequently, BAT activation and WAT/PAT browning hold therapeutic potential for mitigating obesity and T2DM by enhancing systemic insulin sensitivity and reducing metabolic dysfunction(Carrageta et al., 2020; Cheng et al., 2021). Furthermore, BAT differentiation and thermogenic activity are regulated by various biological and environmental factors, including norepinephrine, catecholamines, cold exposure, exercise, overfeeding, increased PGC-1α expression, arousal from hibernation, cancer cachexia, and low-protein diets. Conversely, BAT mass and function are reduced during obesity, prolonged fasting, lactation, T2DM, and hibernation, primarily due to suppressed sympathetic nervous system (SNS) activity, dysregulated insulin signaling, and downregulation of thermogenic genes(Cypess et al., 2015; Harms & Seale, 2013; Trayhurn, 2017; Tsiloulis & Watt, 2015). BAT originates from the somitic mesoderm and differentiates from progenitor cells expressing Eng+ (Endoglin), PAX7+, and Myf5+, which are also derived from dermomyotome precursors. These progenitors share a common lineage with skeletal muscle cells and contribute to myocyte production and skeletal muscle morphology(Ikeda et al., 2018; Sanchez-Gurmaches et al., 2012; Seale et al., 2008; Sebo, Jeffery, Holtrup, & Rodeheffer, 2018). Brown pre-adipocytes secrete key factors, including PRDM16, bone morphogenetic protein 7 (BMP7), PPARγ, PGC-1α, PPARα, Ebf2, and the transcription factor C/EBPβ, which collectively regulate and promote BAT adipogenesis(Harms & Seale, 2013; Seale et al., 2008). Additionally, pharmacological agents such as TZDs and orexin have been shown to enhance BAT development. BMP7 promotes brown adipocyte differentiation by inducing PRDM16 expression, a process further regulated by Ebf2 and PPARγ. Moreover, BMP7 upregulates UCP1 expression, a key thermogenic marker, to enhance BAT function(Tseng et al., 2008). Exercise increases UCP1 expression by approximately 80%, promoting thermogenic activity in BAT. Additionally, exercise enhances the expression of key BAT marker genes, including PRDM16, TBX1, TMEM26, and CD137, which are involved in brown adipocyte differentiation and function(Tsiloulis & Watt, 2015). Additionally, BMP8b, a recently identified regulator of BAT function, primarily enhances BAT sensitivity to sympathetic signaling rather than directly promoting adipogenesis(Whittle et al., 2012). BAT regulates and secretes cytokines, known as BATokines, which mediate autocrine and paracrine signaling. Key BATokines include fibroblast growth factor 21 (FGF21), IL-6, neuregulin 4 (NRG4), and VEGF, all of which contribute to metabolic regulation and tissue remodeling(Chait & den Hartigh, 2020; Lee, Lee, & Oh, 2019). However, adipocyte-derived SCF is dispensable for BAT adipogenesis, indicating distinct regulatory mechanisms for BAT expansion and function(Zhang, Huang, et al., 2019). BAT is characterized by the enriched expression of specific molecular markers. Key transcription factors include LHX8 and ZIC1, which regulate BAT lineage commitment. Additional BAT markers include Eva1, Eps15l1, and Pdk4, as well as microRNAs such as miR-206 and miR-133b, which modulate adipocyte differentiation. Thermogenic and mitochondrial markers include UCP1, CIDEA, PGC-1α, Cox7α, and PRDM16, all of which are essential for BAT function and energy metabolism(Cheng et al., 2021; Harms & Seale, 2013; Ikeda et al., 2018). Additionally, BAT exhibits therapeutic potential in cancer by counteracting browning stimulators such as PTHrP, which has been implicated in cancer-associated cachexia. Given the diverse metabolic functions of BAT, factors that promote brown adipocyte differentiation and induce the browning of WAT hold promise for preventing or mitigating metabolic disorders, including obesity and insulin resistance.

**WAT**

WAT is the most abundant adipose tissue in the human body, comprising approximately 95% of total body fat(Kahn et al., 2019). Its morphology and phenotype differ significantly from those of BAT. WAT consists of spherical adipocytes containing a single large unilocular lipid droplet, with cell size varying depending on lipid storage. Unlike BAT, WAT has fewer mitochondria, which are elongated and less densely packed. In WAT, lipid droplets primarily consist of triglycerides, constituting over 90% of the tissue volume(Cheng et al., 2021; Saely et al., 2012). Unlike BAT, WAT increases with age relative to total body weight. Approximately 80% of WAT is distributed in the subcutaneous region, predominantly beneath the skin and in the abdominal and femoral-gluteal areas (Table S1)(Saely et al., 2012). The remaining WAT is primarily distributed in visceral depots, including the mesenteric and omental fat, as well as adipose tissue surrounding abdominal and retroperitoneal organs and the perigonadal region (Table S1)(D. C. Berry et al., 2013; Bleher et al., 2020; Sanchez-Gurmaches & Guertin, 2014; Tsiloulis & Watt, 2015).

WAT exhibits distinct developmental origins, influencing its cellular and molecular characteristics. Retroperitoneal WAT is derived from the somatic mesoderm, while perigonadal and subcutaneous WAT originate from the lateral plate mesoderm(Sebo et al., 2018). Unlike BAT, which primarily originates from Myf5⁺ mesodermal precursors, WAT is predominantly derived from Myf5⁻ mesodermal progenitors(Cheng et al., 2021). The differentiation of WAT is primarily regulated by the transcription factors PPARγ and CCAAT/enhancer-binding protein alpha (C/EBPα), which play essential roles in adipogenesis. Notably, the expression levels of PPARγ and C/EBPα are higher in subcutaneous WAT compared to visceral WAT(Baglioni et al., 2012; D. C. Berry et al., 2013; Macotela et al., 2012). WAT precursors to include CD24^+^ and Lin^−^ CD29^+^ CD34^+^ Sca-1^+^ CD24^−^ population(R. Berry & Rodeheffer, 2013). Besides, other studies found WAT to derive from APCs characterized with CD31^−^ (non-endothelial cell), and Sca1^+^, CD29^+^ CD34^+^ CD24^+^ population(R. Berry & Rodeheffer, 2013; Bukowska et al., 2018; Rodeheffer, Birsoy, & Friedman, 2008). WAT can also differentiate from perivascular cells, including PCs and VSMCs, which express adipogenic markers. Conversely, WAT-derived adipocytes have been shown to undergo dedifferentiation into dedifferentiated fat cells (DFATCs), which retain multipotency and can further differentiate into VSMCs and PCs. This bidirectional plasticity highlights the dynamic interplay between adipocytes and vascular-associated cells, contributing to tissue remodeling and metabolic adaptability(Zhang, Shao, et al., 2019).

White adipocyte differentiation is regulated by a complex interplay of signaling pathways and transcription factors. Several factors promote WAT adipogenesis, including insulin-like growth factor 1 (IGF-1), insulin, CCAAT/enhancer-binding protein beta (C/EBPβ), and bone morphogenetic proteins (BMP2 and BMP4), which activate downstream transcriptional programs that drive adipocyte commitment and lipid accumulation. Conversely, WAT adipogenesis is inhibited by factors such as transforming growth factor-beta (TGF-β)/SMAD3 signaling, Wnt family member 10B (WNT10B), and GATA-binding transcription factors 2 and 3 (GATA2 and GATA3), which suppress the expression of adipogenic regulators. Additionally, sirtuin signaling pathways, particularly SIRT1 and SIRT3, negatively regulate adipogenesis by modulating mitochondrial function, oxidative metabolism(Cristancho & Lazar, 2011; Reddy et al., 2014).

WAT regulates immune, metabolic, endocrine, and regenerative pathways through intricate cellular and molecular mechanisms(Coelho, Oliveira, & Fernandes, 2013). It is involved in key processes such as energy storage, glucose metabolism, fatty acid mobilization, coagulation, vascular tone regulation, angiogenesis, appetite control, reproduction, and fibrinolysis(Cheng et al., 2021; Fonseca-Alaniz, Takada, Alonso-Vale, & Lima, 2007). Among these processes, WAT primarily functions as a site for lipid storage and can undergo significant hypertrophy, with adipocyte diameters expanding beyond 100 μm, particularly in response to energy surplus rather than fasting(Cheng et al., 2021). It secretes factors such as visfatin, resistin (Retn), leptin, adiponectin, angiotensin, acylation stimulating protein (ASP), sex steroids, glucocorticoids, TNF-α, IL-6, and FFA(Bleher et al., 2020; Fonseca-Alaniz et al., 2007). Unlike BAT, WAT does not contribute directly to thermogenesis. However, through its ability to infiltrate tissues and provide thermal insulation, WAT plays a role in regulating body temperature(Coelho et al., 2013; Saely et al., 2012). WAT may have a significant impact on the tumour microenvironment and vascular inflammation(Correa et al., 2019; Quail & Dannenberg, 2019). For example, excessive WAT expansion creates a hypoxic microenvironment, triggering the release of pro-inflammatory cytokines and pro-angiogenic factors. This inflammatory and angiogenic signaling promotes pathological angiogenesis and may contribute to tumor progression(Correa et al., 2019; Quail & Dannenberg, 2019). Chronic hypoxia may lead to a reduction in marrow adipose tissue (MAT). However, this effect can be reversed by adiponectin, which enhances vascular integrity through the release of anti-inflammatory cytokines (e.g., IL-10) and anti-angiogenic factors (e.g., thrombospondin-1). Additionally, the browning of WAT contributes to an anti-inflammatory response, which modulate immune function and metabolic homeostasis. Overall, WAT plays a crucial role in maintaining metabolic and endocrine homeostasis while serving as an essential energy reservoir during fasting. Notably, its ability to undergo browning, characterized by the upregulation of UCP1 and increased mitochondrial activity, has emerged as a potential therapeutic target for metabolic disorders and cancer treatment.

**BeAT**

Beige adipocytes were recently identified as a distinct adipocyte subtype with characteristics intermediate between WAT and BAT in morphology and function. They are primarily located within WAT depots, including the supraclavicular region (Table S1)(Harms & Seale, 2013). At the cellular level, beige adipocytes contain multilocular lipid droplets and exhibit a mitochondria-rich phenotype, though with fewer mitochondria than classical brown adipocytes(Cheng et al., 2021).

BeAT promotes thermogenesis similarly to BAT; however, unlike BAT, its thermogenic activity can occur through both UCP1-dependent and UCP1-independent mechanisms. The UCP1-independent pathway is primarily regulated by calcium (Ca²⁺) cycling and creatine-driven substrate cycling. Although BeAT expresses UCP1, its expression levels are lower than in BAT but higher than in WAT(Cheng et al., 2021). The overexpression of UCP1 and the resulting increase in thermogenesis enhance glucose uptake, FAO, and lipolysis in BeAT. These metabolic changes improve insulin sensitivity, reduce fat mass, and lower circulating glucose and lipid levels, thereby contributing to the amelioration of metabolic disorders such as T2DM(Cheng et al., 2021). Additionally, in rodents, BeAT plays a crucial role in maintaining body temperature during cold exposure by enhancing thermogenesis(Kajimura, Spiegelman, & Seale, 2015; Zinngrebe et al., 2020).

The origin of BeAT remains a subject of debate, as does its precise role in human physiology, including its contributions to endocrine and metabolic regulation. Beige adipocytes may differentiate from subcutaneous WAT through transdifferentiation, arise from distinct beige adipocyte progenitors, or originate via de novo adipogenesis from tissue-resident progenitor cells(Chait & den Hartigh, 2020; Cheng et al., 2021). Recent studies have identified distinct cellular lineages contributing to BeAT formation. BeAT can originate from precursor cells expressing smooth muscle actin (SMA⁺), platelet-derived growth factor receptor alpha (PDGFR-α⁺), platelet-derived growth factor receptor beta (PDGFR-β⁺), and myogenic factor 5-negative (Myf5⁻) markers(Ikeda et al., 2018). Additionally, lineage-tracing studies in mice have revealed depot-specific differentiation patterns: BeAT in retroperitoneal and subcutaneous WAT can arise from Myf5⁺ progenitors, whereas beige adipocytes in perigonadal and inguinal WAT predominantly differentiate from Myf5⁻ progenitors(Sanchez-Gurmaches & Guertin, 2014; Sanchez-Gurmaches et al., 2012; Seale et al., 2008). These findings suggest that BeAT can arise through multiple differentiation pathways, resulting in distinct marker profiles and subtle functional differences. The conversion of WAT to BeAT is primarily induced by environmental and endogenous factors, including cold exposure, exercise, tissue injury, and pathological conditions such as cancer cachexia. At the molecular level, this process is regulated by key signaling pathways involving peroxisome proliferator-activated receptor gamma coactivator-1 alpha (PGC-1α), PPARγ, and β-adrenergic receptor activation, which drive mitochondrial biogenesis and thermogenic gene expression. Similarly, the absence of key thermogenic regulators leads to the reversal of BeAT into WAT, characterized by the downregulation of UCP1 and a loss of thermogenic capacity(Ikeda et al., 2018). The maintenance and activation of BeAT can also be modulated by the inhibition of specific transcriptional regulators. For instance, the loss of early growth response 1 (Egr1) promotes BeAT differentiation, particularly in inguinal subcutaneous WAT, by increasing the expression of UCP1 and C/EBPβ, both of which are critical for thermogenic gene activation(Zhang, Huang, et al., 2019). Several factors associated with BeAT contribute to the vascularization of adipose tissue. For example, cold exposure and β-adrenergic signaling, particularly through β3-adrenergic receptors, enhance adrenergic stimulation, leading to the upregulation of PGC-1α. This activation subsequently induces the expression of VEGF and its receptor VEGFR2, promoting angiogenesis and improving vascularization in BeAT. This stimulation may also enhance UCP1 expression and thermogenesis, leading to localized acidosis and hypoxia, which activate hypoxia-inducible factor 1-alpha (HIF-1α). HIF-1α, in turn, upregulates vascular endothelial growth factor receptor 2 (VEGFR2) expression(Seki et al., 2016; Sun et al., 2014). VEGFR2 plays a central role in regulating angiogenesis and vasculogenesis by mediating endothelial cell proliferation, migration, and vascular remodeling. In addition to the previously mentioned factors, certain antidiabetic drugs, such as TZDs, can promote BeAT differentiation by binding to PPARγ, which subsequently recruits PRDM16 as a coactivator(Harms & Seale, 2013). BeAT can be identified by the enrichment of specific molecular markers, including Cidea, Pgc1a, Cox7a, PRDM16, Cited1, Cd137, Tmem26, Tmem16, HoxC9, Tbx1, Car4, PAT2, Epsti1, and Dio2, among others(Bukowska et al., 2018; Carey et al., 2014; Garcia, Roemmich, & Claycombe, 2016; Lecka-Czernik et al., 2017).

**PAT**

Unlike other adipocyte subtypes, relatively little is known about the physiology and function of pregnancy-associated adipose tissue, which has been primarily studied in women(Richard, White, Elks, & Stephens, 2000). PAT development is triggered by pregnancy and lactation, during which it is derived from both BAT and WAT through adipocyte remodeling processes(Wang et al., 2018; Zinngrebe et al., 2020). This process is facilitated by the transdifferentiation of milk-secreting alveolar epithelial cells in the mammary gland into adipocytes, contributing to the pink coloration of PAT (Table S1)(Giordano, Smorlesi, Frontini, Barbatelli, & Cinti, 2014; C. Zhou et al., 2016). It exhibits a distinct lipid droplet regulatory profile, predominantly expressing perilipin 2 (Plin2) while lacking Plin1, which contrasts with the expression pattern in WAT. The absence of Plin1 and the enrichment of Plin2 modulate lipid metabolism and have been implicated in attenuating tumor progression by limiting lipid availability for cancer cell proliferation(Apostoli et al., 2014; Cao et al., 2018; C. Zhou et al., 2016). However, PAT may contribute to breast cancer resistance, as the loss of PPARγ during its conversion from WAT disrupts adipocyte differentiation and alters the tumor microenvironment(Apostoli et al., 2014).

**BMAT**

Finally, BMAT, the primary focus of this review, plays a critical role in metabolic regulation and homeostasis through distinct cellular and molecular mechanisms. Although BMAT constitutes only about 10% of total body fat, it resides within the bone marrow microenvironment, where it interacts with HSCs, osteoblasts, and immune cells, influencing hematopoiesis, bone remodeling, and systemic energy metabolism. BMAT-derived adipokines, including adiponectin and leptin, regulate insulin sensitivity, lipid metabolism, and inflammatory responses. Dysregulation of these pathways contributes to the pathogenesis of metabolic disorders, osteoporosis, and hematologic malignancies, making BMAT a promising target for therapeutic intervention.

BMAT is classified into two distinct subtypes: large-sized constitutive BMAT (cBMAT), which develops after birth in distal skeletal sites such as the femur and tibia, and small-sized regulated BMAT (rBMAT), which forms later in life in proximal skeletal regions, including the spinal vertebrae and long bones (Table S1)(Craft, Li, MacDougald, & Scheller, 2018; Krings et al., 2012; Luong, Huang, & Lee, 2019; Scheller et al., 2015; Xia, Mandal, Sinelnikov, Broadhurst, & Wishart, 2012). cBMAT and rBMAT are not only anatomically distinct but also exhibit functional differences. rBMAT is known to play a more dynamic role compared to cBMAT, as it enhances the hematopoietic response to metabolic signals, promotes adipogenesis by supplying adiponectin during aging, provides FFAs to support bone metabolism, and undergoes remodeling in response to various physiological and pathological conditions(Bukowska et al., 2018; Scheller et al., 2019). Recent studies have shown that cBMAT, which histologically resembles WAT, is more resistant to lipolytic stimuli compared to rBMAT and remains relatively unresponsive to metabolic and systemic biological changes(Liu et al., 2013) . Most studies agree that BMAs originate from Myf5⁻ MSCs(Bukowska et al., 2018). Additionally, BM adipocytes share a common Osterix+ (Sp7) skeletal lineage with osteoblasts and chondrocytes, highlighting their developmental plasticity(Chen et al., 2014; Grandl & Wolfrum, 2017; Kir et al., 2014). Adipogenic precursors first commit to a CD45⁻ CD31⁻ Sca1⁻ Zfp423⁺ progenitor state, a step mediated by bone morphogenetic protein (BMP) and Wnt signaling pathways, before terminally differentiating into BMAT adipocytes(Ambrosi et al., 2017; Grandl & Wolfrum, 2017; Tikhonova et al., 2019). Recent studies have identified Leptin-expressing (Lep⁺) MSCs as a key population that differentiates into BMAs, characterized by high expression of matrix Gla protein (MGP) and lipoprotein lipase (LPL)(Tikhonova et al., 2019). Additionally, a novel adipogenic lineage population, termed marrow adipogenic lineage precursors (MALPs). These cells express multiple adipocyte markers and play a crucial role in maintaining bone marrow vasculature but lack significant lipid storage and actively suppress bone formation(Zhong et al., 2020). These findings suggest that BMAT promotes bone marrow vasculature in long bones but not in caudal vertebrae (Table S1)(Mattiucci et al., 2018). The same study identified several molecular markers that are upregulated during the adipogenic differentiation of mesenchymal progenitors, highlighting key regulatory pathways. Transcriptional regulators such as PPARγ and C/EBPα drive adipogenesis, while adipocyte-associated genes, including adiponectin, apolipoprotein E (ApoE), LPL, and leptin receptor (LEPR), modulate lipid metabolism and endocrine signaling. Additionally, inflammatory mediators and extracellular matrix regulators, such as CXCL12, IL1RN, KNG1, KNG2, AGT, ESM1, and GDPD2, contribute to BMAT's interaction with the bone marrow microenvironment. These molecular pathways collectively influence hematopoiesis, angiogenesis, and bone remodeling, positioning BMAT as a key regulator of skeletal and systemic metabolism(Zhong et al., 2020).

Evolutionary analysis and lineage tracing studies have revealed that BMAT shares both similarities and differences with other adipocyte types at the cellular and molecular levels(Suchacki et al., 2020). Morphologically, BMAT resembles WAT; however, it exhibits distinct gene expression patterns, lipid composition, and metabolic activity(Ambrosi et al., 2017; Craft et al., 2018). Unlike BAT and WAT, BMAT is conserved across diverse vertebrate species, including fish, amphibians, reptiles, birds, and mammals(Craft & Scheller, 2017; Gesta et al., 2007). BMAT in the distal tibia exhibits morphological and transcriptional characteristics more similar to inguinal white adipose tissue (iWAT) and gonadal white adipose tissue (gWAT) compared to BMAT in the proximal tibia(Suchacki et al., 2020). At the molecular level, BMAT expresses key adipogenic transcription factors, including PPARγ and C/EBPα, which regulate adipocyte differentiation and lipid metabolism(Ambrosi et al., 2017). Several enriched genes in bone-resident pre-adipocytes are also expressed in brown pre-adipocytes (e.g., Ebf2, Entpd2, Fam129a, and Acy3)(Ambrosi et al., 2017). In addition, BMAT in the tibia expresses BAT-specific gene markers such as PGC-1α, Dio2, PRDM16, but lacks UCP1, which is exclusively expressed in vertebral BMAT. The expression of these thermogenic markers declines with aging and in metabolic disorders such as diabetes(Lindsey & Mohan, 2017; Nishio et al., 2012; Scheller et al., 2015). Several factors may regulate the expression of these thermogenic markers. Treatment with TZDs, such as rosiglitazone—both of which are PPARγ agonists and insulin signaling activators used in diabetes management—did not increase UCP1 expression in tibial BMAT(Sulston et al., 2016). Although, they increased other BAT and WAT markers, besides tri-iodothyronine and thyroid hormone receptor beta- agonist (TRβ) GC-1. They included Pgc1α, Dio2, β3AR, PRDM16 and FoxC2 for BAT, while adiponectin and leptin markers for WAT(Krings et al., 2012; Lindsey & Mohan, 2017; Suchacki & Cawthorn, 2018). From this, BMAT secretes higher levels of adiponectin compared to WAT, suggesting a distinct endocrine role in bone marrow metabolism(Cawthorn et al., 2014; Krings et al., 2012).

The metabolic profile of BMAT is distinct from both BAT and WAT, exhibiting resistance to insulin- and cold-stimulated glucose uptake. In vivo studies have demonstrated that, compared to WAT, BMAT is resistant to insulin-stimulated Akt phosphorylation. Additionally, BMAT exhibits higher expression of SLC2A3 and SLC2A1, while showing reduced expression of SLC2A4 compared to other adipose depots(Suchacki et al., 2020). The SLC2A3 gene encodes glucose transporter 3 (GLUT3), a high-affinity neuronal glucose transporter responsible for facilitating glucose diffusion across the plasma membrane(Ziegler, Almos, McNeill, Jansch, & Lesch, 2020). Thereby, BMAT has higher basal glucose uptake with less insulin sensitiveness(Suchacki et al., 2020). Unlike WAT, BMAT plays a crucial role in bone resorption and remodeling. BMAT promotes the secretion of SCF, which supports HSCs maintenance and expansion, thereby enhancing the production of blood and immune cells(Craft et al., 2018; Krings et al., 2012; B. O. Zhou et al., 2017). It also contributes to leukocyte differentiation and regulates osteoblast development, influencing bone homeostasis and hematopoiesis within the bone marrow microenvironment. BMAT exhibits functional and molecular distinctions from WAT and BAT. BMAT-derived adiponectin and leptin not only regulate adipogenesis but also modulate osteoblast differentiation and bone formation through specific signaling pathways. Meanwhile, RANKL and tumor necrosis factor-alpha (TNF-α) promote osteoclast differentiation and bone resorption. PPARγ plays a critical role in bone-fat balance by regulating sclerostin expression, which in turn induces RANKL production. This pathway contributes to osteoclastogenesis, BMAT adipogenesis, and TZD-induced bone loss, while also influencing bone mass homeostasis by modulating the activity of osteoblasts and osteoclasts(Baroi, Czernik, Chougule, Griffin, & Lecka-Czernik, 2021). BMAT also secretes saturated fatty acids, which inhibit osteoblast formation(Hardouin, Rharass, & Lucas, 2016). Similar to WAT, BMAT also produces anti-inflammatory adipokines, including adiponectin and leptin, which regulate systemic metabolism, immune responses, and bone remodeling(Suchacki et al., 2020). BM stromal cells cultured on 3D silk scaffolds upregulate genes involved in metabolic reprogramming, cell proliferation, and extracellular matrix remodeling, while 2D-cultured cells downregulate genes associated with inflammatory signaling and disease-related pathways. These findings highlight that 3D culture systems better mimic in vivo conditions, providing a more physiologically relevant model for studying BM stromal cell lineage commitment, osteogenic and adipogenic differentiation, and ECM-mediated signaling cascades(Fairfield et al., 2019; Lewis & MacDougald, 2018).

BMAT adipogenesis is regulated by a complex interplay of intrinsic and extrinsic molecular signals. Pro-adipogenic factors, including aging, menopause, estrogen withdrawal, skeletal disuse, alcohol abuse, anorexia nervosa, caloric restriction, glucocorticoids, TZDs, chemotherapy, radiation, obesity, and a high-fat diet, drive BMAT expansion primarily through PPARγ activation, suppression of Wnt/β-catenin signaling, and increased expression of adipogenic transcription factors. Additionally, FGF21, chemerin, secreted frizzled-related protein 1 (Sfrp-1), ghrelin, and sclerostin contribute to BMAT accumulation by modulating BMP signaling, hedgehog pathway inhibition, and adipokine-mediated metabolic regulation. In contrast, anti-adipogenic factors, including exercise, mechanical loading, metformin, vanadate, cold exposure, PTH, and WNT10b, suppress BMAT formation via AMP-activated protein kinase (AMPK) activation, β-adrenergic signaling, and promotion of osteogenic over adipogenic differentiation through Runx2-mediated transcriptional control(Abdullahi et al., 2017; Cawthorn et al., 2012; Cawthorn et al., 2014; Guo et al., 2020; Li, Meng, & Yu, 2019).

These findings suggest that BMAT shares at least some lineage similarities with both BAT and WAT. Emerging insights into the signaling pathways and regulatory mechanisms governing BMAT function within the metabolic and endocrine systems highlight its potential as a therapeutic target for obesity, osteoporosis, and other metabolic disorders.

**Reference:**

Abdullahi, A., Chen, P., Stanojcic, M., Sadri, A. R., Coburn, N., & Jeschke, M. G. (2017). IL-6 Signal From the Bone Marrow is Required for the Browning of White Adipose Tissue Post Burn Injury. Shock, 47(1), 33-39. doi:10.1097/SHK.0000000000000749

Ambrosi, T. H., Scialdone, A., Graja, A., Gohlke, S., Jank, A. M., Bocian, C., . . . Schulz, T. J. (2017). Adipocyte Accumulation in the Bone Marrow during Obesity and Aging Impairs Stem Cell-Based Hematopoietic and Bone Regeneration. Cell Stem Cell, 20(6), 771-784 e776. doi:10.1016/j.stem.2017.02.009

Apostoli, A. J., Skelhorne-Gross, G. E., Rubino, R. E., Peterson, N. T., Di Lena, M. A., Schneider, M. M., . . . Nicol, C. J. (2014). Loss of PPARgamma expression in mammary secretory epithelial cells creates a pro-breast tumorigenic environment. Int J Cancer, 134(5), 1055-1066. doi:10.1002/ijc.28432

Baglioni, S., Cantini, G., Poli, G., Francalanci, M., Squecco, R., Di Franco, A., . . . Luconi, M. (2012). Functional differences in visceral and subcutaneous fat pads originate from differences in the adipose stem cell. PLoS One, 7(5), e36569. doi:10.1371/journal.pone.0036569

Baroi, S., Czernik, P. J., Chougule, A., Griffin, P. R., & Lecka-Czernik, B. (2021). PPARG in osteocytes controls sclerostin expression, bone mass, marrow adiposity and mediates TZD-induced bone loss. Bone, 147, 115913. doi:10.1016/j.bone.2021.115913

Bartelt, A., & Heeren, J. (2014). Adipose tissue browning and metabolic health. Nat Rev Endocrinol, 10(1), 24-36. doi:10.1038/nrendo.2013.204

Berry, D. C., Stenesen, D., Zeve, D., & Graff, J. M. (2013). The developmental origins of adipose tissue. Development, 140(19), 3939-3949. doi:10.1242/dev.080549

Berry, R., & Rodeheffer, M. S. (2013). Characterization of the adipocyte cellular lineage in vivo. Nat Cell Biol, 15(3), 302-308. doi:10.1038/ncb2696

Bleher, M., Meshko, B., Cacciapuoti, I., Gergondey, R., Kovacs, Y., Duprez, D., . . . Havis, E. (2020). Egr1 loss-of-function promotes beige adipocyte differentiation and activation specifically in inguinal subcutaneous white adipose tissue. Sci Rep, 10(1), 15842. doi:10.1038/s41598-020-72698-w

Bukowska, J., Frazier, T., Smith, S., Brown, T., Bender, R., McCarthy, M., . . . Gimble, J. M. (2018). Bone Marrow Adipocyte Developmental Origin and Biology. Curr Osteoporos Rep, 16(3), 312-319. doi:10.1007/s11914-018-0442-z

Cao, Q., Ruan, H., Wang, K., Song, Z., Bao, L., Xu, T., . . . Zhang, X. (2018). Overexpression of PLIN2 is a prognostic marker and attenuates tumor progression in clear cell renal cell carcinoma. Int J Oncol, 53(1), 137-147. doi:10.3892/ijo.2018.4384

Carey, A. L., Vorlander, C., Reddy-Luthmoodoo, M., Natoli, A. K., Formosa, M. F., Bertovic, D. A., . . . Kingwell, B. A. (2014). Reduced UCP-1 content in in vitro differentiated beige/brite adipocytes derived from preadipocytes of human subcutaneous white adipose tissues in obesity. PLoS One, 9(3), e91997. doi:10.1371/journal.pone.0091997

Carrageta, D. F., Oliveira, P. F., Monteiro, M. P., & Alves, M. G. (2020). Adipocyte Specific Signaling. In J. V. Silva, M. J. Freitas, & M. Fardilha (Eds.), Tissue-Specific Cell Signaling (pp. 409-436). Cham: Springer International Publishing.

Cattaneo, P., Mukherjee, D., Spinozzi, S., Zhang, L., Larcher, V., Stallcup, W. B., . . . Guimaraes-Camboa, N. (2020). Parallel Lineage-Tracing Studies Establish Fibroblasts as the Prevailing In Vivo Adipocyte Progenitor. Cell Rep, 30(2), 571-582 e572. doi:10.1016/j.celrep.2019.12.046

Cawthorn, W. P., Bree, A. J., Yao, Y., Du, B., Hemati, N., Martinez-Santibanez, G., & MacDougald, O. A. (2012). Wnt6, Wnt10a and Wnt10b inhibit adipogenesis and stimulate osteoblastogenesis through a beta-catenin-dependent mechanism. Bone, 50(2), 477-489. doi:10.1016/j.bone.2011.08.010

Cawthorn, W. P., Scheller, E. L., Learman, B. S., Parlee, S. D., Simon, B. R., Mori, H., . . . MacDougald, O. A. (2014). Bone marrow adipose tissue is an endocrine organ that contributes to increased circulating adiponectin during caloric restriction. Cell Metab, 20(2), 368-375. doi:10.1016/j.cmet.2014.06.003

Chait, A., & den Hartigh, L. J. (2020). Adipose Tissue Distribution, Inflammation and Its Metabolic Consequences, Including Diabetes and Cardiovascular Disease. Front Cardiovasc Med, 7, 22. doi:10.3389/fcvm.2020.00022

Chen, J., Shi, Y., Regan, J., Karuppaiah, K., Ornitz, D. M., & Long, F. (2014). Osx-Cre targets multiple cell types besides osteoblast lineage in postnatal mice. PLoS One, 9(1), e85161. doi:10.1371/journal.pone.0085161

Cheng, L., Wang, J., Dai, H., Duan, Y., An, Y., Shi, L., . . . Zhao, B. (2021). Brown and beige adipose tissue: a novel therapeutic strategy for obesity and type 2 diabetes mellitus. Adipocyte, 10(1), 48-65. doi:10.1080/21623945.2020.1870060

Chouchani, E. T., Kazak, L., & Spiegelman, B. M. (2019). New Advances in Adaptive Thermogenesis: UCP1 and Beyond. Cell Metab, 29(1), 27-37. doi:10.1016/j.cmet.2018.11.002

Cinti, S. (2009). Transdifferentiation properties of adipocytes in the adipose organ. Am J Physiol Endocrinol Metab, 297(5), E977-986. doi:10.1152/ajpendo.00183.2009

Coelho, M., Oliveira, T., & Fernandes, R. (2013). Biochemistry of adipose tissue: an endocrine organ. Arch Med Sci, 9(2), 191-200. doi:10.5114/aoms.2013.33181

Correa, L. H., Heyn, G. S., & Magalhaes, K. G. (2019). The Impact of the Adipose Organ Plasticity on Inflammation and Cancer Progression. Cells, 8(7). doi:10.3390/cells8070662

Craft, C. S., Li, Z., MacDougald, O. A., & Scheller, E. L. (2018). Molecular differences between subtypes of bone marrow adipocytes. Curr Mol Biol Rep, 4(1), 16-23.

Craft, C. S., & Scheller, E. L. (2017). Evolution of the Marrow Adipose Tissue Microenvironment. Calcif Tissue Int, 100(5), 461-475. doi:10.1007/s00223-016-0168-9

Cristancho, A. G., & Lazar, M. A. (2011). Forming functional fat: a growing understanding of adipocyte differentiation. Nat Rev Mol Cell Biol, 12(11), 722-734. doi:10.1038/nrm3198

Cypess, A. M., Lehman, S., Williams, G., Tal, I., Rodman, D., Goldfine, A. B., . . . Kahn, C. R. (2009). Identification and importance of brown adipose tissue in adult humans. N Engl J Med, 360(15), 1509-1517. doi:10.1056/NEJMoa0810780

Cypess, A. M., Weiner, L. S., Roberts-Toler, C., Franquet Elia, E., Kessler, S. H., Kahn, P. A., . . . Kolodny, G. M. (2015). Activation of human brown adipose tissue by a beta3-adrenergic receptor agonist. Cell Metab, 21(1), 33-38. doi:10.1016/j.cmet.2014.12.009

Fairfield, H., Falank, C., Farrell, M., Vary, C., Boucher, J. M., Driscoll, H., . . . Reagan, M. R. (2019). Development of a 3D bone marrow adipose tissue model. Bone, 118, 77-88. doi:10.1016/j.bone.2018.01.023

Fonseca-Alaniz, M. H., Takada, J., Alonso-Vale, M. I., & Lima, F. B. (2007). Adipose tissue as an endocrine organ: from theory to practice. J Pediatr (Rio J), 83(5 Suppl), S192-203. doi:10.2223/JPED.1709

Garcia, R. A., Roemmich, J. N., & Claycombe, K. J. (2016). Evaluation of markers of beige adipocytes in white adipose tissue of the mouse. Nutr Metab (Lond), 13, 24. doi:10.1186/s12986-016-0081-2

Gesta, S., Tseng, Y. H., & Kahn, C. R. (2007). Developmental origin of fat: tracking obesity to its source. Cell, 131(2), 242-256. doi:10.1016/j.cell.2007.10.004

Giordano, A., Smorlesi, A., Frontini, A., Barbatelli, G., & Cinti, S. (2014). White, brown and pink adipocytes: the extraordinary plasticity of the adipose organ. Eur J Endocrinol, 170(5), R159-171. doi:10.1530/EJE-13-0945

Giralt, M., & Villarroya, F. (2013). White, brown, beige/brite: different adipose cells for different functions? Endocrinology, 154(9), 2992-3000. doi:10.1210/en.2013-1403

Grandl, G., & Wolfrum, C. (2017). Adipocytes at the Core of Bone Function. Cell Stem Cell, 20(6), 739-740. doi:10.1016/j.stem.2017.05.008

Guo, Y., Huo, J., Wu, D., Hao, H., Ji, X., Zhao, E., . . . Liu, Q. (2020). Simvastatin inhibits the adipogenesis of bone marrow‑derived mesenchymal stem cells through the downregulation of chemerin/CMKLR1 signaling. Int J Mol Med, 46(2), 751-761. doi:10.3892/ijmm.2020.4606

Hardouin, P., Rharass, T., & Lucas, S. (2016). Bone Marrow Adipose Tissue: To Be or Not To Be a Typical Adipose Tissue? Front Endocrinol (Lausanne), 7, 85. doi:10.3389/fendo.2016.00085

Harms, M., & Seale, P. (2013). Brown and beige fat: development, function and therapeutic potential. Nat Med, 19(10), 1252-1263. doi:10.1038/nm.3361

Ikeda, K., Maretich, P., & Kajimura, S. (2018). The Common and Distinct Features of Brown and Beige Adipocytes. Trends Endocrinol Metab, 29(3), 191-200. doi:10.1016/j.tem.2018.01.001

Kahn, C. R., Wang, G., & Lee, K. Y. (2019). Altered adipose tissue and adipocyte function in the pathogenesis of metabolic syndrome. J Clin Invest, 129(10), 3990-4000. doi:10.1172/JCI129187

Kajimura, S., Spiegelman, B. M., & Seale, P. (2015). Brown and Beige Fat: Physiological Roles beyond Heat Generation. Cell Metab, 22(4), 546-559. doi:10.1016/j.cmet.2015.09.007

Kir, S., White, J. P., Kleiner, S., Kazak, L., Cohen, P., Baracos, V. E., & Spiegelman, B. M. (2014). Tumour-derived PTH-related protein triggers adipose tissue browning and cancer cachexia. Nature, 513(7516), 100-104. doi:10.1038/nature13528

Krings, A., Rahman, S., Huang, S., Lu, Y., Czernik, P. J., & Lecka-Czernik, B. (2012). Bone marrow fat has brown adipose tissue characteristics, which are attenuated with aging and diabetes. Bone, 50(2), 546-552. doi:10.1016/j.bone.2011.06.016

Lecka-Czernik, B., Stechschulte, L. A., Czernik, P. J., Sherman, S. B., Huang, S., & Krings, A. (2017). Marrow Adipose Tissue: Skeletal Location, Sexual Dimorphism, and Response to Sex Steroid Deficiency. Front Endocrinol (Lausanne), 8, 188. doi:10.3389/fendo.2017.00188

Lee, M. W., Lee, M., & Oh, K. J. (2019). Adipose Tissue-Derived Signatures for Obesity and Type 2 Diabetes: Adipokines, Batokines and MicroRNAs. J Clin Med, 8(6). doi:10.3390/jcm8060854

Lewis, K. T., & MacDougald, O. A. (2018). Bone: Bone marrow adipocytes in 3D. Nat Rev Endocrinol, 14(5), 254-255. doi:10.1038/nrendo.2018.31

Li, Y., Meng, Y., & Yu, X. (2019). The Unique Metabolic Characteristics of Bone Marrow Adipose Tissue. Front Endocrinol (Lausanne), 10, 69. doi:10.3389/fendo.2019.00069

Lidell, M. E., Betz, M. J., & Enerback, S. (2014). Brown adipose tissue and its therapeutic potential. J Intern Med, 276(4), 364-377. doi:10.1111/joim.12255

Lindsey, R. C., & Mohan, S. (2017). Thyroid hormone acting via TRbeta induces expression of browning genes in mouse bone marrow adipose tissue. Endocrine, 56(1), 109-120. doi:10.1007/s12020-017-1265-x

Liu, Y., Strecker, S., Wang, L., Kronenberg, M. S., Wang, W., Rowe, D. W., & Maye, P. (2013). Osterix-cre labeled progenitor cells contribute to the formation and maintenance of the bone marrow stroma. PLoS One, 8(8), e71318. doi:10.1371/journal.pone.0071318

Luong, Q., Huang, J., & Lee, K. Y. (2019). Deciphering White Adipose Tissue Heterogeneity. Biology (Basel), 8(2). doi:10.3390/biology8020023

Macotela, Y., Emanuelli, B., Mori, M. A., Gesta, S., Schulz, T. J., Tseng, Y. H., & Kahn, C. R. (2012). Intrinsic differences in adipocyte precursor cells from different white fat depots. Diabetes, 61(7), 1691-1699. doi:10.2337/db11-1753

Mattiucci, D., Maurizi, G., Izzi, V., Cenci, L., Ciarlantini, M., Mancini, S., . . . Poloni, A. (2018). Bone marrow adipocytes support hematopoietic stem cell survival. J Cell Physiol, 233(2), 1500-1511. doi:10.1002/jcp.26037

Neamat-Allah, J., Wald, D., Husing, A., Teucher, B., Wendt, A., Delorme, S., . . . Kaaks, R. (2014). Validation of anthropometric indices of adiposity against whole-body magnetic resonance imaging--a study within the German European Prospective Investigation into Cancer and Nutrition (EPIC) cohorts. PLoS One, 9(3), e91586. doi:10.1371/journal.pone.0091586

Nishio, M., Yoneshiro, T., Nakahara, M., Suzuki, S., Saeki, K., Hasegawa, M., . . . Saeki, K. (2012). Production of functional classical brown adipocytes from human pluripotent stem cells using specific hemopoietin cocktail without gene transfer. Cell Metab, 16(3), 394-406. doi:10.1016/j.cmet.2012.08.001

Pant, R., Firmal, P., Shah, V. K., Alam, A., & Chattopadhyay, S. (2020). Epigenetic Regulation of Adipogenesis in Development of Metabolic Syndrome. Front Cell Dev Biol, 8, 619888. doi:10.3389/fcell.2020.619888

Prokesch, A., Smorlesi, A., Perugini, J., Manieri, M., Ciarmela, P., Mondini, E., . . . Cinti, S. (2014). Molecular aspects of adipoepithelial transdifferentiation in mouse mammary gland. Stem Cells, 32(10), 2756-2766. doi:10.1002/stem.1756

Quail, D. F., & Dannenberg, A. J. (2019). The obese adipose tissue microenvironment in cancer development and progression. Nat Rev Endocrinol, 15(3), 139-154. doi:10.1038/s41574-018-0126-x

Raajendiran, A., Ooi, G., Bayliss, J., O'Brien, P. E., Schittenhelm, R. B., Clark, A. K., . . . Watt, M. J. (2019). Identification of Metabolically Distinct Adipocyte Progenitor Cells in Human Adipose Tissues. Cell Rep, 27(5), 1528-1540 e1527. doi:10.1016/j.celrep.2019.04.010

Reddy, N. L., Tan, B. K., Barber, T. M., & Randeva, H. S. (2014). Brown adipose tissue: endocrine determinants of function and therapeutic manipulation as a novel treatment strategy for obesity. BMC Obes, 1, 13. doi:10.1186/s40608-014-0013-5

Richard, A. J., White, U., Elks, C. M., & Stephens, J. M. (2000). Adipose Tissue: Physiology to Metabolic Dysfunction. In K. R. Feingold, B. Anawalt, M. R. Blackman, A. Boyce, G. Chrousos, E. Corpas, W. W. de Herder, K. Dhatariya, K. Dungan, J. Hofland, S. Kalra, G. Kaltsas, N. Kapoor, C. Koch, P. Kopp, M. Korbonits, C. S. Kovacs, W. Kuohung, B. Laferrère, M. Levy, E. A. McGee, R. McLachlan, M. New, J. Purnell, R. Sahay, A. S. Shah, F. Singer, M. A. Sperling, C. A. Stratakis, D. L. Trence, & D. P. Wilson (Eds.), Endotext. South Dartmouth (MA): MDText.com, Inc.

Copyright © 2000-2025, MDText.com, Inc.

Rodeheffer, M. S., Birsoy, K., & Friedman, J. M. (2008). Identification of white adipocyte progenitor cells in vivo. Cell, 135(2), 240-249. doi:10.1016/j.cell.2008.09.036

Saely, C. H., Geiger, K., & Drexel, H. (2012). Brown versus white adipose tissue: a mini-review. Gerontology, 58(1), 15-23. doi:10.1159/000321319

Sanchez-Gurmaches, J., & Guertin, D. A. (2014). Adipocyte lineages: tracing back the origins of fat. Biochim Biophys Acta, 1842(3), 340-351. doi:10.1016/j.bbadis.2013.05.027

Sanchez-Gurmaches, J., Hung, C. M., Sparks, C. A., Tang, Y., Li, H., & Guertin, D. A. (2012). PTEN loss in the Myf5 lineage redistributes body fat and reveals subsets of white adipocytes that arise from Myf5 precursors. Cell Metab, 16(3), 348-362. doi:10.1016/j.cmet.2012.08.003

Scheller, E. L., Doucette, C. R., Learman, B. S., Cawthorn, W. P., Khandaker, S., Schell, B., . . . MacDougald, O. A. (2015). Region-specific variation in the properties of skeletal adipocytes reveals regulated and constitutive marrow adipose tissues. Nat Commun, 6, 7808. doi:10.1038/ncomms8808

Scheller, E. L., Khandaker, S., Learman, B. S., Cawthorn, W. P., Anderson, L. M., Pham, H. A., . . . MacDougald, O. A. (2019). Bone marrow adipocytes resist lipolysis and remodeling in response to beta-adrenergic stimulation. Bone, 118, 32-41. doi:10.1016/j.bone.2018.01.016

Seale, P., Bjork, B., Yang, W., Kajimura, S., Chin, S., Kuang, S., . . . Spiegelman, B. M. (2008). PRDM16 controls a brown fat/skeletal muscle switch. Nature, 454(7207), 961-967. doi:10.1038/nature07182

Sebo, Z. L., Jeffery, E., Holtrup, B., & Rodeheffer, M. S. (2018). A mesodermal fate map for adipose tissue. Development, 145(17). doi:10.1242/dev.166801

Sebo, Z. L., & Rodeheffer, M. S. (2019). Assembling the adipose organ: adipocyte lineage segregation and adipogenesis in vivo. Development, 146(7). doi:10.1242/dev.172098

Seki, T., Hosaka, K., Lim, S., Fischer, C., Honek, J., Yang, Y., . . . Cao, Y. (2016). Endothelial PDGF-CC regulates angiogenesis-dependent thermogenesis in beige fat. Nat Commun, 7, 12152. doi:10.1038/ncomms12152

Shimizu, I., Aprahamian, T., Kikuchi, R., Shimizu, A., Papanicolaou, K. N., MacLauchlan, S., . . . Walsh, K. (2014). Vascular rarefaction mediates whitening of brown fat in obesity. J Clin Invest, 124(5), 2099-2112. doi:10.1172/JCI71643

Suchacki, K. J., & Cawthorn, W. P. (2018). Molecular Interaction of Bone Marrow Adipose Tissue with Energy Metabolism. Curr Mol Biol Rep, 4(2), 41-49. doi:10.1007/s40610-018-0096-8

Suchacki, K. J., Tavares, A. A. S., Mattiucci, D., Scheller, E. L., Papanastasiou, G., Gray, C., . . . Cawthorn, W. P. (2020). Bone marrow adipose tissue is a unique adipose subtype with distinct roles in glucose homeostasis. Nat Commun, 11(1), 3097. doi:10.1038/s41467-020-16878-2

Sulston, R. J., Learman, B. S., Zhang, B., Scheller, E. L., Parlee, S. D., Simon, B. R., . . . Cawthorn, W. P. (2016). Increased Circulating Adiponectin in Response to Thiazolidinediones: Investigating the Role of Bone Marrow Adipose Tissue. Front Endocrinol (Lausanne), 7, 128. doi:10.3389/fendo.2016.00128

Sun, K., Kusminski, C. M., Luby-Phelps, K., Spurgin, S. B., An, Y. A., Wang, Q. A., . . . Scherer, P. E. (2014). Brown adipose tissue derived VEGF-A modulates cold tolerance and energy expenditure. Mol Metab, 3(4), 474-483. doi:10.1016/j.molmet.2014.03.010

Tikhonova, A. N., Dolgalev, I., Hu, H., Sivaraj, K. K., Hoxha, E., Cuesta-Dominguez, A., . . . Aifantis, I. (2019). The bone marrow microenvironment at single-cell resolution. Nature, 569(7755), 222-228. doi:10.1038/s41586-019-1104-8

Trayhurn, P. (2017). Origins and early development of the concept that brown adipose tissue thermogenesis is linked to energy balance and obesity. Biochimie, 134, 62-70. doi:10.1016/j.biochi.2016.09.007

Trayhurn, P. (2022). Brown Adipose Tissue: A Short Historical Perspective. Methods Mol Biol, 2448, 1-18. doi:10.1007/978-1-0716-2087-8_1

Tseng, Y. H., Kokkotou, E., Schulz, T. J., Huang, T. L., Winnay, J. N., Taniguchi, C. M., . . . Kahn, C. R. (2008). New role of bone morphogenetic protein 7 in brown adipogenesis and energy expenditure. Nature, 454(7207), 1000-1004. doi:10.1038/nature07221

Tsiloulis, T., & Watt, M. J. (2015). Exercise and the Regulation of Adipose Tissue Metabolism. Prog Mol Biol Transl Sci, 135, 175-201. doi:10.1016/bs.pmbts.2015.06.016

Virtanen, K. A., Lidell, M. E., Orava, J., Heglind, M., Westergren, R., Niemi, T., . . . Nuutila, P. (2009). Functional brown adipose tissue in healthy adults. N Engl J Med, 360(15), 1518-1525. doi:10.1056/NEJMoa0808949

Wang, Q. A., Song, A., Chen, W., Schwalie, P. C., Zhang, F., Vishvanath, L., . . . Scherer, P. E. (2018). Reversible De-differentiation of Mature White Adipocytes into Preadipocyte-like Precursors during Lactation. Cell Metab, 28(2), 282-288 e283. doi:10.1016/j.cmet.2018.05.022

Whittle, A. J., Carobbio, S., Martins, L., Slawik, M., Hondares, E., Vazquez, M. J., . . . Vidal-Puig, A. (2012). BMP8B increases brown adipose tissue thermogenesis through both central and peripheral actions. Cell, 149(4), 871-885. doi:10.1016/j.cell.2012.02.066

Wu, J., Bostrom, P., Sparks, L. M., Ye, L., Choi, J. H., Giang, A. H., . . . Spiegelman, B. M. (2012). Beige adipocytes are a distinct type of thermogenic fat cell in mouse and human. Cell, 150(2), 366-376. doi:10.1016/j.cell.2012.05.016

Xia, J., Mandal, R., Sinelnikov, I. V., Broadhurst, D., & Wishart, D. S. (2012). MetaboAnalyst 2.0--a comprehensive server for metabolomic data analysis. Nucleic Acids Res, 40(Web Server issue), W127-133. doi:10.1093/nar/gks374

Zhang, Z., Huang, Z., Ong, B., Sahu, C., Zeng, H., & Ruan, H. B. (2019). Bone marrow adipose tissue-derived stem cell factor mediates metabolic regulation of hematopoiesis. Haematologica, 104(9), 1731-1743. doi:10.3324/haematol.2018.205856

Zhang, Z., Shao, M., Hepler, C., Zi, Z., Zhao, S., An, Y. A., . . . Scherer, P. E. (2019). Dermal adipose tissue has high plasticity and undergoes reversible dedifferentiation in mice. J Clin Invest, 129(12), 5327-5342. doi:10.1172/JCI130239

Zhong, L., Yao, L., Tower, R. J., Wei, Y., Miao, Z., Park, J., . . . Qin, L. (2020). Single cell transcriptomics identifies a unique adipose lineage cell population that regulates bone marrow environment. Elife, 9. doi:10.7554/eLife.54695

Zhou, B. O., Yu, H., Yue, R., Zhao, Z., Rios, J. J., Naveiras, O., & Morrison, S. J. (2017). Bone marrow adipocytes promote the regeneration of stem cells and haematopoiesis by secreting SCF. Nat Cell Biol, 19(8), 891-903. doi:10.1038/ncb3570

Zhou, C., Wang, M., Zhou, L., Zhang, Y., Liu, W., Qin, W., . . . Tang, J. (2016). Prognostic significance of PLIN1 expression in human breast cancer. Oncotarget, 7(34), 54488-54502. doi:10.18632/oncotarget.10239

Ziegler, G. C., Almos, P., McNeill, R. V., Jansch, C., & Lesch, K. P. (2020). Cellular effects and clinical implications of SLC2A3 copy number variation. J Cell Physiol, 235(12), 9021-9036. doi:10.1002/jcp.29753

Zinngrebe, J., Debatin, K. M., & Fischer-Posovszky, P. (2020). Adipocytes in hematopoiesis and acute leukemia: friends, enemies, or innocent bystanders? Leukemia, 34(9), 2305-2316. doi:10.1038/s41375-020-0886-x
